# Cnot8 eliminates naïve regulation networks and is essential for naïve-to-formative pluripotency transition

**DOI:** 10.1093/nar/gkac236

**Published:** 2022-04-07

**Authors:** Yujun Quan, Meijiao Wang, Chengpeng Xu, Xiaoxiao Wang, Yu Wu, Dandan Qin, Yuxuan Lin, Xukun Lu, Falong Lu, Lei Li

**Affiliations:** State Key Laboratory of Stem Cell and Reproductive Biology, Institute of Stem Cell and Regeneration, Beijing Institute of Stem Cell and Regenerative Medicine, Institute of Zoology, Chinese Academy of Sciences, Beijing 100101, China; University of Chinese Academy of Sciences, Beijing 100049, China; State Key Laboratory of Molecular Developmental Biology, Institute of Genetics and Developmental Biology, Innovative Academy of Seed Design, Chinese Academy of Sciences, Beijing 100101, China; State Key Laboratory of Stem Cell and Reproductive Biology, Institute of Stem Cell and Regeneration, Beijing Institute of Stem Cell and Regenerative Medicine, Institute of Zoology, Chinese Academy of Sciences, Beijing 100101, China; University of Chinese Academy of Sciences, Beijing 100049, China; State Key Laboratory of Stem Cell and Reproductive Biology, Institute of Stem Cell and Regeneration, Beijing Institute of Stem Cell and Regenerative Medicine, Institute of Zoology, Chinese Academy of Sciences, Beijing 100101, China; State Key Laboratory of Stem Cell and Reproductive Biology, Institute of Stem Cell and Regeneration, Beijing Institute of Stem Cell and Regenerative Medicine, Institute of Zoology, Chinese Academy of Sciences, Beijing 100101, China; University of Chinese Academy of Sciences, Beijing 100049, China; State Key Laboratory of Stem Cell and Reproductive Biology, Institute of Stem Cell and Regeneration, Beijing Institute of Stem Cell and Regenerative Medicine, Institute of Zoology, Chinese Academy of Sciences, Beijing 100101, China; State Key Laboratory of Stem Cell and Reproductive Biology, Institute of Stem Cell and Regeneration, Beijing Institute of Stem Cell and Regenerative Medicine, Institute of Zoology, Chinese Academy of Sciences, Beijing 100101, China; State Key Laboratory of Stem Cell and Reproductive Biology, Institute of Stem Cell and Regeneration, Beijing Institute of Stem Cell and Regenerative Medicine, Institute of Zoology, Chinese Academy of Sciences, Beijing 100101, China; State Key Laboratory of Molecular Developmental Biology, Institute of Genetics and Developmental Biology, Innovative Academy of Seed Design, Chinese Academy of Sciences, Beijing 100101, China; University of Chinese Academy of Sciences, Beijing 100049, China; State Key Laboratory of Stem Cell and Reproductive Biology, Institute of Stem Cell and Regeneration, Beijing Institute of Stem Cell and Regenerative Medicine, Institute of Zoology, Chinese Academy of Sciences, Beijing 100101, China; University of Chinese Academy of Sciences, Beijing 100049, China

## Abstract

Mammalian early epiblasts at different phases are characterized by naïve, formative, and primed pluripotency states, involving extensive transcriptome changes. Here, we report that deadenylase Cnot8 of Ccr4-Not complex plays essential roles during the transition from naïve to formative state. Knock out (KO) *Cnot8* resulted in early embryonic lethality in mice, but *Cnot8* KO embryonic stem cells (ESCs) could be established. Compared with the cells differentiated from normal ESCs, *Cnot8* KO cells highly expressed a great many genes during their differentiation into the formative state, including several hundred naïve-like genes enriched in lipid metabolic process and gene expression regulation that may form the naïve regulation networks. Knockdown expression of the selected genes of naïve regulation networks partially rescued the differentiation defects of *Cnot8* KO ESCs. *Cnot8* depletion led to the deadenylation defects of its targets, increasing their poly(A) tail lengths and half-life, eventually elevating their expression levels. We further found that Cnot8 was involved in the clearance of targets through its deadenylase activity and the binding of Ccr4-Not complex, as well as the interacting with Tob1 and Pabpc1. Our results suggest that Cnot8 eliminates naïve regulation networks through mRNA clearance, and is essential for naïve-to-formative pluripotency transition.

## INTRODUCTION

During developmental progression from blastocyst to gastrulation embryos, plentiful hallmark events occur in the pluripotent epiblast between embryonic day (E) 4.0 and E8.0 in mice. Pluripotent stem cells (PSCs) isolated from inner cell mass (ICM) of mouse blastocyst are known as embryonic stem cells (ESCs) (1,2), while PSCs derived from mouse post-implantation embryos around E5.5-E8.0 are named as epiblast stem cells (EpiSCs) (3,4). Mouse ESCs maintained in 2i/LIF medium are similar to pre-implantation epiblast cells at E4.5, and regarded as a naïve state that is not ready for cell differentiation (5), while EpiSCs are close to mouse anterior primitive streak cells at ∼E7.5, and are thought of as a primed state that is biased for cell commitment (6). Accumulating evidence implies the presence of intermediate states between naïve and primed states of PSCs (7–10). In fact, RSCs (rosette-like stem cells) were recently reported as an intermediate state between naïve and primed states (11). We and other two groups have established other cell lines with the formative features between naïve and primed states, designated as fPSCs (formative pluripotent stem cells), FS (formative stem) cells, and XPSCs [chimera (Xιμαιρα in Greek) and PGC dual-competent PSCs] (12–14). fPSCs and FS cells are close to ∼E5.5–6.5 epiblast cells, while XPSCs and RSCs are similar to the epiblast cells at ∼E5.0 (15,16). From naïve to formative state, epiblast cells dramatically transform the epigenomic state and metabolic program to prepare their transcriptome and signaling for the cell lineage specification during gastrulation. Thus, the investigation of naïve-to-formative transition is important for the understanding of epiblast development and direct differentiation of stem cells.

Specific kinase inhibitors (2i) of MEK/ERK and GSK3 signaling pathway facilitate ESC self-renewal and maintain high expression levels of core transcription factor (TF) *Oct4*, *Sox2*, and *Nanog* (17–19). *Oct4* couples with *Sox2* and *Nanog* to positively modulate their own expression, forming a core autoregulatory circuitry that governs the naïve pluripotent state of ESCs (20). These core TFs also function together to promote the expression of actively transcribed genes including many naïve TFs that are essential for sustaining ground state, such as *Klf2/4/5*, *Esrrb*, *Nr5a2*, *Tbx3*, *Jam2*, and *Tfcp2l1*, by binding super-enhancers and/or recruiting coactivators to their promoters and RNA polymerase II (21–23). The core and naïve TFs may constitute the basis of gene regulatory networks (GRNs) to maintain ESC self-renewal and naïve pluripotency state (17,24–26). The GRNs have been used to characterize the naïve state of ESCs with computational approaches, accelerating the understanding of pluripotency (27,28). After release from the inhibition of MEK/ERK and GSK3 signaling pathway, ESCs exit from naïve state and become a formative phase at specific culture conditions (7,8,12,14). Compared with ESCs, formative PSCs express low levels of many naïve-like genes, including many naïve TFs of GRNs (12,14), suggesting a degradation of the naïve-like genes and GRNs during naïve-to-formative state transition. These observations indicate that RNA degradation plays important roles in the formative progression from naïve pluripotent state. Nevertheless, how RNA degradation functions in this transition remains poorly understood.

In most organisms from yeast to metazoan, mRNA degradation or translation suppression is initiated by the deadenylation or shortening of mRNA poly(A) tails that is often the rate-limiting step. Ccr4-Not (carbon catabolite repression 4-negative on TATA-less) complex is evolutionarily conserved and a major machinery of deadenylation in eukaryotic cells (29). This complex is mainly consisted of the regulatory module including the scaffold protein Not1 and the deadenylase module (30). The physiological functions of multiprotein Ccr4-Not complex are very complicated in organisms. For example, Caf1p and Ccr4p are the only two deadenylases of yeast Ccr4-Not complex, however, disruption of both genes results in mild phenotypes that display no worse growth than those in the single mutants of these genes, suggesting that the roles of these two proteins are compensated by other enzymes (31). Although Not1p is the only protein essential for yeast viability, deletion of *Cnot1* or *Cnot3* results in mouse embryonic lethality (32,33). Consistent with their essential roles in mouse early embryonic development, *Cnot1* or *Cnot3* has been shown to be required for self-renewal of mouse and human ESCs (34–36). Accumulating evidence suggests that the missense mutations in components of Ccr4-Not complex are associated with various human diseases. For instance, the mutations in CNOT1 are related to syndrome of pancreatic agenesis and holoprosencephaly (HPE) (37,38). CNOT3 mutations are also linked to T-cell acute lymphoblastic leukemia (T-ALL) (39) and ventricular tachyarrhythmias (32).

As an essential machinery in organisms, Ccr4-Not complex plays important roles in numerous biological processes, such as transcription elongation and translation efficiency, and is best known as a regulator in mRNA deadenylation and subsequent decay (31,33). Due to coexistence of highly similar deadenylase orthologs of Ccr4 (Cnot6 and Cnot6l) and Caf1 (Cnot7 and Cnot8) in the complex, its role of mRNA deadenylation is more intricate in mammals (40). A good deal of evidence suggests that mammalian Ccr4-Not complex regulates mRNA deadenylation and decay in different cell contexts by these four deadenylases. For example, Cnot6l promotes the deadenylation and degradation of a subset of mRNAs in mouse oocyte maturation (41,42). Cnot6 has also been implicated in the deadenylation of *Slbp* and *Orc6* mRNAs during maturation of mouse oocytes (43). On the other hand, due to ∼81% identity of amino acids in their DEDD domains, the roles of Caf1 ortholog Cnot7 and Cnot8 in deadenylation may be compensated and more complicated. Actually, both Cnot7 and Cnot8 have been shown to possess similar activity of deadenylation *in vitro* and to be involved in the same pathway in many cells (44,45). In mice, both Cnot7 and Cnot8 of Ccr4-Not complex are involved in the deadenylation and decay of mRNAs through Btg4 (B-cell translocation gene 4), a member of Btg and Tob (Transducer of ErbB2) family and a key regulator of mouse maternal-to-zygotic transition (46–48). In addition, human CNOT7 and CNOT8 have been shown to function redundantly in MCF7 cell propagation, which has been shown to be dependent on their deadenylase activity (49). Nevertheless, how these deadenylases of Ccr4-Not complex function in mammalian PSC fate transition remains unknown.

In this study, we found that depletion of Cnot8, but not other deadenylases impaired the transition of PSCs from naïve to formative pluripotency state. Disruption of *Cnot8* resulted in the stabilization and the increased poly(A) tail lengths of mRNAs of a great many genes including ∼300 naïve GRN-like genes, such as *Nanog*, *Tbx3*, *Esrrb*, and *Klf2*, during the transition from naïve to formative state in mouse PSCs. The defects of cell fate transition could be partially restored by eliminating *Nanog*, *Tbx3*, or *Klf2* with shRNA in *Cnot8*-deleted ESCs. Cnot8 modulated the poly(A) tail lengths of target mRNAs and was involved in mRNA clearance through its deadenylase activity, the interactions of Ccr4-Not complex, as well as Tob1 and Pabpc1. These findings suggest that Cnot8 deadenylates the poly(A) tail of its targets, subsequently degrading the mRNAs of naïve GRN genes, and is essential for the formative progression from naïve pluripotency state.

## MATERIALS AND METHODS

### Animals

All ICR strain mice used in this study were maintained under Specific Pathogen Free (SPF) conditions with a standard 12:12 light: dark cycle. All experimental procedures were implemented in compliance with the guidelines of Animal Care and Use Committee of the Institute of Zoology, Chinese Academy of Sciences (CAS).

### Cell culture

Mouse embryonic stem cells (ESCs) were cultured on feeder-free petri dishes or plates coated with 0.2% gelatin (Millipore, 901771) in 2i/LIF medium at 37°C under 5% CO_2_ as we previously reported (50). N2B27 medium contains 48% DMEM/F12 (Gibco, 11330032), 48% Neurobasal Medium (Gibco, 21103049), 0.5% N2 supplement (Invitrogen, 17502048), 1% B27 supplement (Invitrogen, 17504044), 1% GlutaMAX (Invitrogen, 35050061), 1% MEM non-essential amino acids (NEAA, Gibco, 11140050), 1% penicillin-streptomycin (Invitrogen, 15140122), 1% sodium pyruvate (Invitrogen, 11360070), 5 mg/ml bovine serum albumin (BSA, Sigma-Aldrich, A4378), and 0.1 mM β-mercaptoethanol (Sigma-Aldrich, M6250). The 2i/LIF medium for ESCs contains 1,000 U/ml recombinant human LIF (Millipore, ESG1107), 1 μM PD0325901 (LC Laboratories, P-9688), and 3 μM CHIR99021 (LC Laboratories, C-6556) in N2B27 medium. HEK293FT cells were purchased from the American Type Culture Collection (ATCC) and cultured in DMEM basic (Gibco, C11995500BT) containing 10% fetal bovine serum (FBS, PAN, ST30-3302) at 37°C under 5% CO_2_.

For cell passage and differentiation, the cells were prewashed twice with DPBS (Sigma-Aldrich, D5652), added with 0.25% Trypsin-EDTA (Invitrogen, 25200072) in the petri dishes, and incubated for 1 min at 37°C. Then, the cells were added with equal volume of FBS (Millipore, ES-009-B) and collected by centrifuge at 1,000 g for 3 min for the experiments. Mouse embryonic fibroblasts (MEFs) from ICR strain mice were inactivated by mitomycin C (Sigma-Aldrich, M0503) and used as the feeder cells. For all experiments, mycoplasma contamination was routinely pretested in the cells.

### Generation of mutant mice and ESC lines using CRISPR/Cas9

Single guide RNA (sgRNA) targeting the exon 2 of *Cnot6*, *Cnot6l*, or *Cnot8*, or the exon 4 of *Cnot7* was designed with the website of MIT CRISPR design (http://cripsr.mit.edu/). *Cas9* mRNAs and sgRNAs were *in vitro* synthesized from p$ \times$330 vector (Addgene) according to the procedures previously described (51). For superovulation, normal ICR females of 6–8 weeks were interperitoneally injected with 5 IU of pregnant mare serum gonadotropin (PMSG, Ningbo Second Hormone Factory,) and 5 IU of human chorionic gonadotropin (HCG, Ningbo Second Hormone Factory) (46–48 h after PMSG injection). After collected from the fertilized females, the zygotes were injected with the mixture of 100 ng/μl *Cas9* mRNA and 50 ng/μl sgRNA, and cultured in KSOM medium for another 3–4 h at 37°C under 5% CO_2_. Then, the embryos were implanted into the oviducts of surrogate mothers to produce offspring. F0 male chimeras were crossed with normal ICR females for germline transmission. The offspring were genotyped by the PCR with their genomic DNA with specific primers. Wild-type (WT) alleles for *Cnot6/6l* were identified with *Cnot6/6l*-P2 and *Cnot6/6l*-P3 primer. *Cnot6/6l*-P1 and *Cnot6/6l*-P3 primers were used to identify *Cnot6/6l* mutant. *Cnot7/8* WT alleles were genotyped with *Cnot7/8*-P1 and *Cnot7/8*-P2 primer. *Cnot7/8*-P1 and *Cnot7/8*-P3 were utilized for the identification of *Cnot7/8* mutant.

To generate *Cnot7* or *Cnot8*-depleted ESC lines, mouse E3.5 blastocysts were harvested from the mating of *Cnot7* or *Cnot8* heterozygote females and males, and transferred to MEFs-coated 3.5 cm petri dishes containing 15% Knockout Serum Replacement (KSR, Gibco, 10828028) medium. The culture medium contained 15% KSR, 1$ \times$penicillin-streptomycin, 2 mM GlutaMAX, 0.1 mM MEM non-essential amino acid (NEAA), 1 mM sodium pyruvate, 0.1 mM β-mercaptoethanol, and 2i/LIF (1 μM PD0325901, 3 μM CHIR99021, and 1,000 units/ml LIF). After 48 h of culture, the medium was replaced with fresh 2i/LIF medium. Single colony was manually picked for propagation. ESCs were genotyped through PCR with specific primers and confirmed through Western blot with specific antibodies. All sequences of primers and sgRNAs for *Cno6*, *Cnot6l*, *Cnot7*, and *Cnot8* were listed in Supplementary Table S1.

### Dissection of post-implantation embryos

Mouse post-implantation embryos at embryonic day (E) E6.5 (12:00), E7.5 (12:00), E8.5 (12:00), E9.5 (12:00), and E13.5 (12:00) were obtained from the mating of *Cnot8* heterozygote females and males. The extra-embryonic tissues were manually removed. The recovered embryos were washed with PBS and imaged with microscope. Nested PCR was used for the genotyping of embryos. Each embryo was lysed with 20 μl buffer A (25 mM NaOH, 0.2 mM EDTA, pH 8.0) and 20 μl buffer B (40 mM Tris-HCl, pH 8.0), and incubated for 50 min at 95°C. The primers of *Cnot8*-P1/*Cnot8*-seq-R were utilized for the first round PCR, and *Cnot8*-seq-F/*Cnot8*-seq-R primer was used for the second PCR. PCR products were confirmed through Sanger sequencing with *Cnot8*-seq-F primer. All primers used were listed in Supplementary Table S1.

### *In vitro* culture (IVC) of mouse embryos

*In vitro* culture (IVC) assay of mouse embryos was carried out according to the procedures that we previously described (52). Briefly, the mouse blastocysts (E3.5) were recovered from the mating of *Cnot8* heterozygote females and males, transferred to 3.5 cm petri dishes precoated with Matrigel (BD, BD356230), and cultured in CMRL 1066 medium (Gibco, 11530037) containing 10% FBS (Millipore, ES-009-B), 1 mM sodium pyruvate, 100 units/ml penicillin-streptomycin, 2 mM L-Glutamine (Gibco, 25030081) at 37°C under 5% CO_2_ for the first two days (IVC day 1–2). When the embryos were solidly attached onto the petri dishes, the culture medium contained 20% FBS was used. After culture for additional 2–3 days, the embryos developed into egg cylinder-like stage were used for the next experiments. For genotyping the IVC embryos, nested PCR and Sanger sequencing were used. All primers used were listed in Supplementary Table S1.

### Plasmid construction and generation of stable ESC lines

Full-length (FL) cDNAs of mouse *Cnot8*, *Cnot7*, and *Tob1* were amplified from the cDNA of normal ESCs through PCR with KOD-Plus-Neo (TOYOBO, KOD-401), and were subcloned into pHIV-EGFP vector (Addgene) with a 3$ \times$FLAG tag at HpaI site. Cnot8 point-mutant proteins, including 3$ \times$FLAG-pHIV-EGFP-Cnot8 D40A/D42A (Mut1), 3$ \times$FLAG-pHIV-EGFP-Cnot8 E138K/D149K/T142Y (Mut2), and 3$ \times$FLAG-pHIV-EGFP-Cnot8 E247A/Y260A (Mut3), were achieved by an overlap site-directed mutagenesis method. All primers were used for plasmid construction as listed in Supplementary Table S1.

Cnot8, Cnot7, and Tob1 FL, and Cnot8 D40A/D42A (Mut1), E138K/D149K/T142Y (Mut2), and E247A/Y260A (Mut3) were subcloned into 3$ \times$FLAG-pHIV-EGFP vector. Lentivirus was produced through co-transfection of individual vector with psPAX2 (Addgene) and pMD2.G (Addgene) in a 4:3:2 ratio with HEK293FT cells using polyetherimide (PEI) (Sigma-Aldrich, 61128–46-9) according to the manufacturer's instructions. The supernatant containing lentivirus particles was harvested at 72 h post-transfection and filtered with a 0.22 μm filter. WT or *Cnot8*-deleted ESCs were seeded in the 24-well plates precoated with gelatin and infected with the supernatant of lentivirus in the presence of 8 μg/ml polybrene (Sigma-Aldrich, H9268). After 8–12 h infection, the culture medium was replaced with fresh 2i/LIF medium. GFP^+^ colonies were manually picked and propagated for generation of stable ESC lines. The cell lines with overexpression genes were verified through Western blot with specific antibodies.

### Formation of RSCs, XPSCs, EpiLCs, FS cells, and fPSCs from mouse ESCs

RSCs, XPSCs, FS cells, and fPSCs were induced and maintained from mouse naïve ESCs under the specific conditions as previously reported (11–14). Epiblast-like cell (EpiLC) formation was performed as previously described with minor modifications (8). Briefly, ESCs cultured in 2i/LIF medium were prewashed with DPBS and dissociated into single cells with 0.25% Trypsin-EDTA. 1$ \times$10^5^ cells were seeded in the 12-well plate precoated with a 1:100 mixture of Matrigel and DMEM/F12, and supplemented with EpiLC formation medium containing N2B27, 20 ng/ml Activin A (PeproTech, 120–14), 12 ng/ml bFGF2 (R&D Systems, 233-FB), and 1% KSR. During EpiLC formation, the culture medium was replaced every day.

### Alkaline phosphatase staining

Alkaline phosphatase (AP) staining for ESCs and EpiLCs was implemented using the BCIP/NBT Alkaline Phosphatase Color Development Kit (Beyotime, C3206) according to the manufacturer's instructions. Briefly, the cells were rinsed twice with PBS and fixed with 4% paraformaldehyde (PFA, Sigma-Aldrich, 158127) for 10 min at room temperature. After rinsed twice with PBST, the cells were stained with BCIP/NBT solution for 30 min at room temperature. The images were captured with microscope.

### Reverse ESC (rESC) formation

After dissociating, 1$ \times$10^4^ EpiLCs of each genotype were seeded into the 12-well plate coated with MEFs, and cultured in 2i/LIF medium for 4 days. Then, the cells were rinsed twice with PBS. AP staining was performed using the BCIP/NBT Alkaline Phosphatase Color Development Kit according to the manufacturer's instructions. Colonies were imaged and analyzed in 6–8 random fields under a $ \times$2 microscope.

### Hematoxylin and eosin (H&E) staining and immunostaining

Natural embryos were dissected from the fertilized females at E7.5 and E9.5. The embryos with maternal tissues were washed with PBS, fixed in 4% PFA for 24 h at 4°C, embedded in paraffin, and sliced into 8 μm sections. The H&E staining was carried out for the paraffin sections according to the standard protocol. The images of H&E staining were captured with the Leica Aperio VERSA 8 microscope (Leica Biosystems).

For immunostaining, ESCs, RSCs, XPSCs, EpiLCs, and fPSCs were fixed with 4% PFA for 15 min (20 min for IVC embryos) at room temperature, washed three times with 0.1% Triton $ \times$-100 in PBS, and permeabilized with 1% Triton $ \times$-100 in PBS for 15 min (30 min for IVC embryos). After blocked with 1% BSA in PBS for 30 min at room temperature, the cells were incubated with primary antibodies overnight at 4°C. For *in vivo* embryo staining, the embryos were fixed in 4% PFA for 20 min at room temperature. After washed with 0.1% Triton $ \times$-100 in PBS, the embryos were permeabilized and blocked with 0.3% Triton $ \times$-100 and 0.1% Glycine in PBS for 30 min at room temperature. Then, the embryos were incubated with primary antibodies diluted with 10% FBS (PAN) and 1% Tween-20 in PBS overnight at 4°C. After three times of washes with 0.1% Triton $ \times$-100 in PBS, the cells and embryos were incubated with the secondary antibodies conjugated with fluorescence and counterstained with Hoechst 33342 (Sigma-Aldrich, B2261) for 1 h at room temperature. The images were captured with Laser scanning confocal microscopes LSM 780 (Carl Zeiss), LSM 880 (Carl Zeiss), and LSM 880 Fast Ariyscan (Carl Zeiss). All antibodies were listed in Supplementary Table S2.

### Western blot and protein quantification

Cells were pre-rinsed twice with ice-cold DPBS and lysed for 40 min on ice with RIPA buffer [5 mM Tris-HCl, pH7.5, 150 mM NaCl, 1% Triton $ \times$-100, 1% sodium deoxycholate, 0.1% sodium dodecyl sulfate (SDS), 1 mM Na_3_VO_4_, 5 mM EDTA, and 10 mM NaF] containing 1$ \times$complete EDTA-free protease inhibitor cocktail (Roche). The concentration of protein was determined with BCA Protein Assay Kit (Beyotime, P0012). The samples were boiled for 5 min at 95°C, separated in 8–12% SDS-PAGE gels, and transferred to polyvinylidene fluoride (PVDF) membranes (Millipore, IPVH00010). After blocked with TBST containing 5% (w/v) defatted milk for 1 h at room temperature, the membranes were incubated with specific primary antibodies overnight at 4°C. After washed with PBST, the membranes were incubated with second antibodies. Western blot signal was developed with SuperSignal^TM^ West Pico PLUS Chemiluminescent Substrate (Thermo Scientific, 34580) and SuperSignal^TM^ West Femto Maximum Sensitivity Substrate (Thermo Scientific, 34095). The band intensity of target protein was determined using Image Lab Software (Bio-Rad) and Image J software (Media Cybernetics). All antibodies for Western blot were listed in Supplementary Table S2.

### RNA isolation, reverse transcription, and quantitative PCR (qRT-PCR)

Total RNA and mRNA were extracted from the cells using RNAzol Reagent (Molecular Research Center, RN190) following the manufacturer's instructions. To avoid the contamination of genomic DNA, total RNA was treated with RQ1 RNase-Free DNase (Promega, M610A) for 30 min at 37°C and purified using the phenol-chloroform extraction and ethanol precipitation. mRNA was extracted from the IVC embryos with the Dynabeads^TM^ mRNA DIRECT^TM^ Micro kit (Invitrogen, 61021) according to the manufacturer's instructions. The concentration of RNA was determined by NanoDrop 2000 (Thermo Fisher Scientific). The cDNA was synthesized with PrimeScript^TM^ RT Reagent kit (Perfect Real Time) (Takara Bio, RR037A) containing Oligo(dT) and Random Primers with 1 μg RNA per sample. qRT-PCR was carried out with EvaGreen 2$ \times$qPCR MasterMix-No Dye (ABM, MasterMix-S) on LightCycler 480 II (Roche). mRNA abundances of target mRNAs were normalized to the internal control of *Gapdh* using a 2^–ΔΔCt^ method with at least three independent experiments. All primers for qRT-PCR were listed in Supplementary Table S1.

### RNA interference assay

To stably knock down the expressions of specific genes in *Cnot8* KO ESCs, a lentiviral Tet-inducible system of EZ-Tet-pLKO-Puro plasmid (Addgene) was used as previously described (53). Briefly, shRNA construct for *Nanog*, *Klf2*, or *Tbx3* was designed to target 19 or 21 base-pair of the specific coding region of these genes (54,55). Oligonucleotides were subcloned into the EZ-Tet-pLKO-Puro plasmid that was predigested with NheI and EcoR I. Lentivirus was produced through co-transfection of the vectors with psPAX2 and pMD2.G in a ratio of 4:3:2 into HEK293FT cells using PEI according to the manufacturer's instructions. Supernatant containing lentivirus was harvested after 72 h transfection and filtered with a 0.22 μm filter. *Cnot8* KO ESCs were seeded in the 24-well plate precoated with gelatin, and infected with the supernatant of lentivirus in the presence of 8 μg/ml polybrene. At 12 h of post-infection, the cells were cultured in fresh 2i/LIF medium. After puromycin (1 μg/ml) selection for 4 days, 8–10 independent colonies were manually picked and expanded for RNA interference assay. The efficiency of interference was validated by qRT-PCR after the treatment with doxycycline (2 μg/ml) for 72 h.

For generation of the stable ESC lines of *Pabpc1* or *Cnot1* knockdown, shRNA construct for *Pabpc1* or *Cnot1* was designed to target 21 base-pair of the specific coding region of these genes. Oligonucleotides were subcloned into the EZ-Tet-pLKO-Puro plasmid that was predigested with NheI and EcoRI. All shRNA oligonucleotides were listed in Supplementary Table S1.

### mRNA stability analysis

2.5$ \times$10^5^ cells were seeded in the 3.5 cm petri dishes that were precoated with gelatin. After culture for 48 h, the cells were treated with 5 μM actinomycin D (Sigma-Aldrich, A4262) to inhibit RNA transcription and were harvested at 0, 2, or 4 h, respectively. Total RNA was isolated for qRT-PCR analysis with the primers of specific genes.

The stability or half-life of mRNA was calculated according to the procedure previously described (56). After turning off transcription of mRNA with the treatment of actinomycin D, the change rate of mRNA quantity at a given time (*dC/dt*) was proportional to both the mRNA decay rate (*K_decay_*) and the quantity of remaining cytoplasmic mRNA (*C*), resulting in the following formula:

$$\begin{equation*}dC/dt = - {K_{decay}}C\end{equation*}$$

### The rate *K_decay_* of mRNA degradation was calculated by

$$\begin{equation*}ln\left( {C/{C_0}} \right) = - {K_{decay}}t\end{equation*}$$

Where *C_0_* is the quantity of mRNA at time 0 h.

To determine the mRNA half-life (*t_1/2_*) that 50% of the existing mRNA was degraded (that is, *C*/*C_0_= 1/2*), the formula was performed as follows:

$$\begin{equation*}\ln \left( {1/2} \right) = - {K_{decay}}{t_{1/2}}\end{equation*}$$

From where:

$$\begin{equation*}{t_{1/2}} = \ln 2/{K_{decay}}\end{equation*}$$

All primers for qRT-PCR were listed in Supplementary Table S1.

### Coimmunoprecipitation (Co-IP) and RIP assay

ESCs were rinsed twice with prechilled DPBS and lysed in Cell lysis buffer of Western and IP (Beyotime) containing 1$ \times$complete EDTA-free protease inhibitor cocktail. Before the Co-IPs, the cells were treated with 20 μg/ml RNase A for 40 min at 37°C and incubated for 40 min on ice. After centrifugation at 12,000 rpm for 15 min at 4°C, 5% of supernatant was saved as ‘input’, and the rests were incubated with Protein A/G Magnetic Beads (MedChemExpress, HY-K0202; Bimake, B23202) and the antibody against FLAG (Sigma-Aldrich, F1804) or negative control IgG (Abclonal, AC011; Santa Cruz Biotechnology, sc2025) overnight at 4°C with gentle rotation. After washing 5 times with lysis buffer, the beads were added with 1$ \times$sodium dodecyl sulphate (SDS) loading buffer and boiled for 5 min at 95°C. The samples were analyzed with Western blot using the specific antibodies.

RNA immunoprecipitation (RIP) assay was implemented following the method previously described with mild modifications (41). Briefly, FLAG-Cnot8 overexpressed ESCs were cultured in 6 cm petri dishes. After rinsed twice with prechilled DPBS, the cells were harvested with 1 ml RIP lysis buffer [50 mM Tris, pH 7.4, 150 mM NaCl, 1% Triton $ \times$-100, 5 mM EDTA, 1$ \times$complete EDTA-free protease inhibitor cocktail, and 200 U/ml RNase inhibitor (Thermo Fisher Scientific, EO0381)]. The cell lysates were incubated for 40 min on ice and centrifuged at 12,000 rpm for 15 min at 4°C. Part of the supernatants was saved as ‘input’, the rests were incubated with Protein A/G Magnetic Beads (MedChemExpress, HY-K0202) prewashed with RIP lysis buffer, and the antibody against FLAG (Sigma-Aldrich, F1804) or negative control IgG (Abclonal, AC011). After incubating for 4 h at 4°C with gentle rotation, the complex including magnetic beads-antibody-protein-RNA was washed 5 times with the washing buffer [50 mM Tris (pH 7.4), 500 mM NaCl, 0.1% Triton $ \times$-100, 5 mM EDTA, 1$ \times$complete EDTA-free protease inhibitor cocktail, and 200 U/ml RNase inhibitor], and subjected to extraction of mRNA using RNAzol Reagent according to the manufacturer's protocols. cDNA synthesis was performed using PrimeScript^TM^ RT Reagent kit (Perfect Real Time). The mRNA abundances were analyzed by RT-PCR. All primers for RT-PCR were listed in Supplementary Table S1.

### Poly(A) test (PAT) assay

The length of poly(A) tail of each transcript was determined as previously described with minor modifications (57). Briefly, to produce the Oligo(dT)-anchored P primer, a GC-rich anchor sequence PAT-P (5′-GCGAGCTCCGCGGCCGCGT_12_-3′) and the 5′-end of Oligo(dT) were treated with T4 DNA ligase (Thermo Fisher, EL0011) for 2 h at 20°C. 500 ng of total RNA was used for reverse transcription using the SuperScript II Reverse Transcriptase (Invitrogen, 18064–014) and Oligo(dT)-anchored P primer. PAT-PCR was performed using gene-specific forward primer (GSP-F) and Oligo(dT)-anchored P or gene-specific reverse (A0) primer (GSP-R) with KOD-Plus-Neo. PCR products were subjected to electrophoresis in a 2% agarose gel and stained with GelRed dye. All primers for PAT-PCR were listed in Supplementary Table S1.

### RNA-seq, scRNA-seq, and PAIso-seq

Total RNA was extracted from ESCs and EpiLCs at different time points using RNAzol Reagent following the manufacturer's instructions. To avert the contamination of genomic DNA, total RNA was treated with RQ1 RNase-Free DNase for 30 min at 37°C and purified with phenol-chloroform extraction and ethanol precipitation. 3 μg of total RNA was used for the library construct according to the manufacturer's instructions (Illumina). RNA sequencing was performed using the Illumina Hiseq $ \times$-Ten platform with pair end 150 bp (PE150). The replicates of each condition were used for sequencing.

For scRNA-seq, WT (*ROSA^mT/mG^*) and *Cnot8* KO ESCs (S/LIF) and 48h-EpiLCs were dissociated into single cells with TrypLE Express (Gibco, 12605010) for 1 min at 37°C, and equal volume of FBS was used for inactivation of TrypLE Express. The cells were centrifuged at 1,000g for 3 min, washed twice with DPBS, and passed through a 40 μm strainer. Then, the cells were resuspended in 0.04% BSA in DPBS and counted. Equal numbers of WT or *Cnot8* KO ESCs (S/LIF) or 48h-EpiLCs were mixed. After suspending, the samples were immediately placed on ice for the preparation of gel bead in emulsion (GEM) for reverse transcription. The 10$ \times$Genomics-based single-cell RNA sequencing (scRNA-seq) libraries were constructed using the Chromium™ Single Cell 3′ Reagent Kits v2 protocol (CG00052). Sequencing was implemented on an Illumina NovaSeq 6000 platform with pair end 150 bp (PE150).

Genome-wide poly(A) tail features on each transcript were determined as a method that we previously described with minor modifications (58). Briefly, total RNA from *Cnot8* KO and control cells at 24 h after EpiLC formation was extracted using RNAzol Reagent according to the manufacturer's instructions, and stored at −80°C or used immediately. 500 ng of total RNA was subjected to barcoded end extension, template switching, full-length cDNA amplification, and circular adapter ligation. PAIso-seq was performed to precisely decipher the lengths and base compositions of RNA poly(A) tails using the circular full-length cDNA libraries on the PacBio third-generation sequencing platform. The replicates for each condition were barcoded with spiking-ins and sequenced. All primers used for PAIso-seq library construction were listed in Supplementary Table S1.

### Sequencing data processing

Raw paired-end reads with low quality and adaptors were trimmed with Cutadapt software (version 0.3.8). Clean reads were mapped to the mouse genome (mm9) using TopHat2 (version 2.1.1) with default parameters. The expression levels of each transcript were determined with the gene annotation of UCSC RefSeq database using FeatureCounts (version 2.0.1) and were presented as transcripts per million (TPM). Differentially expressed genes (DEGs) were identified [log_2_ TPM > 1, fold change (FC) > 1.5] by comparing expression level of each gene in KO cells with those in normal controls. Expression patterns of specific genes across the different time points of normal ESC differentiation were generated using the Markov clustering algorithm and were visualized as boxplots. Heatmaps of the changed gene expression were clustered with Hierarchical clustering analysis and were visualized with the TreeView software (version 1.1.6r4). To gain overall insights into progression of cell fate transition between KO and WT cells, principal component analysis (PCA) was carried out and the results were visualized using all expressed genes (http://www.r-project.org), and the importance of each dimension was also calculated. GO term analysis for the enriched biological processes were conducted using the web tool: The Database for Annotation, Visualization and Integrated Discovery (DAVID) (https://david.ncifcrf.gov/) with the default parameters.

For scRNA-seq data analysis, raw data from two mixed samples based on 10$ \times$Genomics were processed by Cell Ranger software suite (version 3.0.2) with default parameters using the mm10 reference genome (Cell ranger reference version 2020). The standard reference genome was modified to incorporate with the coding sequence (CDS) of *tdTomato* gene. The Seurat package (version 4) was used to analyze the data. Principal component analysis (PCA) was performed for dimension reduction and *t*-distributed stochastic neighbor embedding (t-SNE) was further used to project the cells into two dimensions. The transcriptional noise between the cells was analyzed as previously reported (59,60).

For PAIso-seq data processing, circular consensus sequence (CCS) reads were extracted from raw subreads and the sequence errors were corrected by using ccs (version 4.2.0) with default parameters. The extracted CCS reads were split into different samples with barcodes and were further trimmed to remove the adapters. The processed CCS reads were transformed to standard FASTQ files and were mapped to mouse genome (mm10) using minimap2 (version 2.17) with the parameters: -ax splice -uf – secondary = no – MD – cs. After moved repetitive reads, the aligned bam files from minimap2 were parsed and the soft-clip nucleotide were extracted as the candidates of primary 3′poly(A) tail sequence. For high-quality poly(A) tails, we filtered out the sequences with less than 4 As at the first 8 bp. To focus on the shortening of poly(A) tails, we discarded the transcripts without poly(A) tails (3′ soft clip = 0). We extended upstream continuous ‘A’-tracts in alignments if it was necessary for the purpose of misalignment. We assigned transcripts with the defined poly(A) tail to specific genes with FeatureCounts (version 2.0.1). The ultimate trimmed sequence of the CCS reads was regarded as the poly(A) tails of each transcript. The poly(A) tail length of transcript was calculated as the length of the trimmed sequence. Since the distribution of poly(A) tail length of each gene is a lognormal-like distribution, the poly(A) tail lengths of genes were presented with the geometric mean of the poly(A) tail lengths of transcripts from the same gene. For poly(A) tail length analysis, we compared the global poly(A) tail lengths of all transcripts with the transcripts from the assigned genes at the same time. For global comparison, CCS transcripts with well-defined poly(A) tail sequence were retained and plotted for the length distributions with passes no less than seven if they were not designated to make sure the accuracy of poly(A) tail length and base calling. To make statistical comparison, we filtered the genes with less than 10 transcripts and calculated the geometric mean to stand for gene poly(A) tail lengths.

### Statistical analysis

All statistical analyses were performed with GraphPad Prism 7 (GraphPad software, Inc). Statistical significance of differences was determined using two-tailed Student's *t* test as described in figure legend. Data were represented as mean ± SEM. A value of ρ < 0.05 was regarded as statistical significance.

## RESULTS

### *Cnot8* is essential for mouse early embryonic development

**Figure 1.**
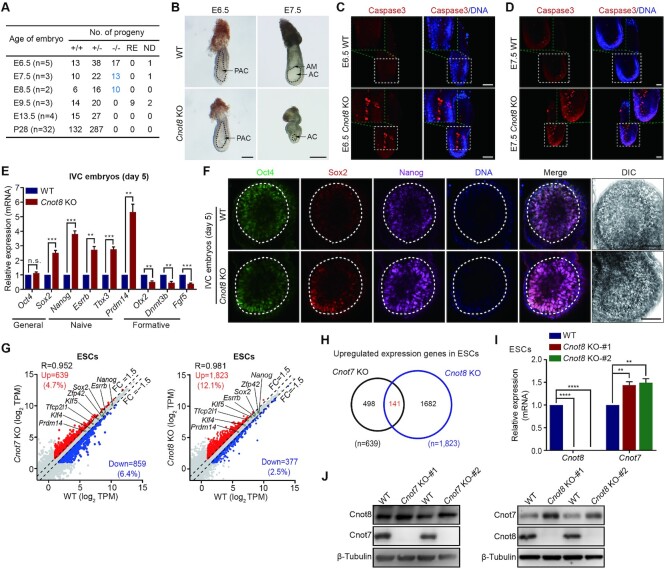
*Cnot8* is essential for mouse early embryonic development. (**A**) Genotyping of *Cnot8^+/+^*, *Cnot8^+/–^*, and *Cnot8^–/–^* embryos and offspring. The numbers of *Cnot8^–/–^* embryos that displayed smaller size and malformed morphology at E7.5 to E8.5 were marked in light blue. Notably, no *Cnot8 ^–/–^* embryos was recovered at and after E9.5. RE, resorbed embryos; ND, not determined; E, embryonic day; P, postnatal day; n, number of litters of embryos. (**B**) Representative bright-field images of WT and *Cnot8* KO embryos at E6.5 and E7.5. WT, wild type; KO, Knockout; PAC, pro-amniotic cavity; AC, amniotic cavity; AM, amnion. Black dashed lines indicated the PAC (lefty panel) and AC (right pane). Scale bars, 100 μm (left panel) and 250 μm (right panel). (**C**, **D**) Cleaved Caspase-3 immunostaining analysis of WT and *Cnot8* KO natural embryos at E6.5 (**C**) and E7.5 (**D**). Whole mount staining was performed for these embryos with the antibody for Cleaved Caspase-3 (red). DNA was counterstained with Hoechst 33342 (blue). Insets were higher magnification images of boxed regions. Scale bar, 50 μm. (**E**) Quantitative RT-PCR (qRT-PCR) analysis of the expression of key pluripotent genes in WT and *Cnot8* KO IVC egg cylinder-like embryos that were *in vitro* cultured for 5 days from the blastocysts. Data were represented as mean ± SEM, n = 3 biological replicates. **ρ < 0.01, ***ρ < 0.001, ns, not significant by two-tailed Student's *t* test. IVC, *in vitro* culture. (**F**) Immunostaining analysis of Oct4, Sox2 and Nanog for WT and *Cnot8* KO IVC embryos. Blastocysts were cultured *in vitro* for 5 days and developed into egg cylinder-like stage. Whole mount staining was performed for these IVC embryos with the antibodies for Oct4 (green), Sox2 (red), and Nanog (purple). DNA was counterstained with Hoechst 33342 (blue). Bright-field images were treated with DIC. White dashed lines indicated epiblast cells. Scale bar, 50 μm. (**G**) Scatter plots showing expression patterns of genes in WT, *Cnot7* KO, and *Cnot8* KO naïve ESCs. The genes whose expressions were upregulated or downregulated more than 1.5-fold in KO cells compared with the controls were highlighted in red and blue, respectively. Representative general and naïve pluripotent genes were also shown according to the expression levels in WT, *Cnot7* KO, and *Cnot8* KO ESCs. (**H**) The numbers of genes in Venn diagram showing intersection of the genes whose expressions were upregulated in both *Cnot7* and *Cnot8* KO ESCs when compared with normal controls. (**I**) qRT-PCR analysis of *Cnot7* mRNA in WT and *Cnot8* KO ESCs. Data were represented as mean ± SEM, n = 3 biological replicates. **ρ < 0.01, ****ρ < 0.0001 by two-tailed Student's *t* test. (**J**) Western blot analysis of Cnot8 and Cnot7 proteins in WT, *Cnot7* KO, and *Cnot8* KO ESCs. β-Tubulin served as a loading control.

To investigate the physiological roles of deadenylases in Ccr4-Not complex that were differentially expressed in mouse tissues (Supplementary Figure S1A), we generated knockout (KO) mouse strains for *Cnot6*, *Cnot6l*, *Cnot7*, and *Cnot8* using CRISPR/Cas9 system (Supplementary Figure S1B). The genotype of mice and embryos were analyzed using PCR with the specific primers for *Cnot6*, *Cnot6l*, *Cnot7*, and *Cnot8* (Supplementary Figure S1C). *Cnot6^–^^/^^–^*, *Cnot6l^–^^/^^–^*, and *Cnot7^–^^/^^–^* mice grew to adulthood without obvious defects, whereas no *Cnot8*^–/–^ mice was born from the mating of heterozygous males and females (Figure 1A and Supplementary Figure S1C), in accord with the previous reports (61,62). To investigate the phenotype of *Cnot8* mutant mice, we dissected the embryos from the mating of heterozygote males and females, and genotyped these embryos with PCR and sequencing. Consistent with the expected Mendelian ratios, the embryos with different genotype were obtained before embryonic day 9.5 (E9.5), whereas no *Cnot8*^–/–^ embryos was acquired at and after E9.5 (Figure 1A). Compared with the controls (wild type, WT), *Cnot8* KO embryos exhibited the similar size and morphology with the formation of egg cylinder at E6.5, whereas they displayed smaller size and malformed morphology at E7.5 (Figure 1A and B). H&E staining revealed that *Cnot8* depletion resulted in the smaller amniotic cavity at E7.5 and yolk sac at E9.5 when compared with the WT controls (Supplementary Figure S1D and E). Cleaved Caspase-3 immunostaining suggested that apoptosis barely occurred in WT controls, whereas severe apoptosis took place in *Cnot8* KO embryos at E6.5 and E7.5 (Figure 1C and D).

Due to the difficult accessibility of peri-implantation embryos, blastocysts from heterozygote mating were cultured with an *in vitro* culture (IVC) system (52). Mouse blastocysts (98 in total for 3 experiments) from the intercrosses of *Cnot8* heterozygous males and females were cultured in Matrigel-coated petri dishes. After 5-day culture, 47 embryos developed into egg cylinder-like stage with a typical cup-shaped structure and the genotype of 11 embryos were authenticated as *Cnot8* homozygotes. The successful ratio of IVC embryos at egg cylinder-like stage was ∼48.0% (47/98), comparable to our previous report (52). The ratio of homozygotes at egg cylinder-like stage was ∼23.4% (11/47) in all cultured embryos, close to the Mendel ratio. These results suggest that disruption of *Cnot8* did not obstruct the structure formation of egg cylinder of mouse embryos.

To investigate the defects of *Cnot8* KO embryos at molecular levels, we examined mRNA expression of key developmental regulators with quantitative RT-PCR (qRT-PCR) in the IVC KO embryos. Compared with WT controls, *Cnot8* KO embryos expressed significantly high levels of general (*Sox2*) and naïve transcription factors (TFs) (*Nanog*, *Esrrb*, *Tbx3*, and *Prdm14)*, but low levels of formative genes (*Otx2*, *Dnmt3b*, and *Fgf5)* (Figure 1E). The expressions of trophoblast and extraembryonic endoderm (XEN) markers were also downregulated in *Cnot8* KO IVC embryos, probably consistent with the malformed amnion and yolk sac after *Cnot8* deficiency (Supplementary Figure S1D-F). We also stained the IVC embryos with the specific antibodies for Oct4, Sox2, and Nanog. Our results showed that the signals of Sox2 and Nanog, but not Oct4 staining were stronger in *Cnot8* KO embryos than those in WT controls (Figure 1F). Collectively, these results indicate that *Cnot8* is critical for mouse early embryonic development.

### *Cnot8* is required for the differentiation of naïve ESCs into formative state.

To investigate the roles and mechanisms of Ccr4-Not complex in the mRNA deadenylation of epiblast development, we established mouse ESC lines from *Cnot7* or *Cnot8* KO embryos (Supplementary Figure S2A). Depletion of *Cnot7* or *Cnot8* did not interfere the alkaline phosphatase (AP) activity, nor the expression abundances of Oct4, Sox2, and Nanog in mouse ESCs (Supplementary Figure S2B-E). Then, we performed RNA sequencing (RNA-seq) for WT, *Cnot7* KO, and *Cnot8* KO ESCs. Unexpectedly, despite little effect on mouse embryonic development and ESC growth, disruption of *Cnot7* led to the upregulated expression of 639 genes and downregulated expression of 859 genes in *Cnot7* KO ESCs (Figure 1G). We also identified 1,823 upregulated and 377 downregulated genes in *Cnot8* KO ESCs (Figure 1G and Supplementary Table S3-4). Although the expression levels of large numbers of genes changed in *Cnot7* KO (up and down, ∼11.1%) or *Cnot8* KO (up and down, ∼14.6%) ESCs, most key developmental regulators were expressed at the similar levels in *Cnot7* or *Cnot8* KO cells when compared with the controls (Figure 1G), suggesting that depletion of *Cnot7* or *Cnot8* barely affects the naïve state of mouse ESCs. Compared with the controls, 141 genes with upregulated expression were overlapped in *Cnot7* and *Cnot8* KO ESCs, suggesting the common targets of *Cnot7* and *Cnot8* (Figure 1H). Notably, the expression of *Cnot7* was significantly increased in *Cnot8* KO ESCs at mRNA and protein levels when compared with WT controls, similar to the changes of Cnot8 mRNA and protein in *Cnot7* KO ESCs (Figure 1I-J and Supplementary Figure S2F). In addition, we could not establish the double KO ESCs for *Cnot7* and *Cnot8* (data not shown). These results support the compensatory roles of *Cnot8* and *Cnot7* in mouse ESCs.

**Figure 2.**
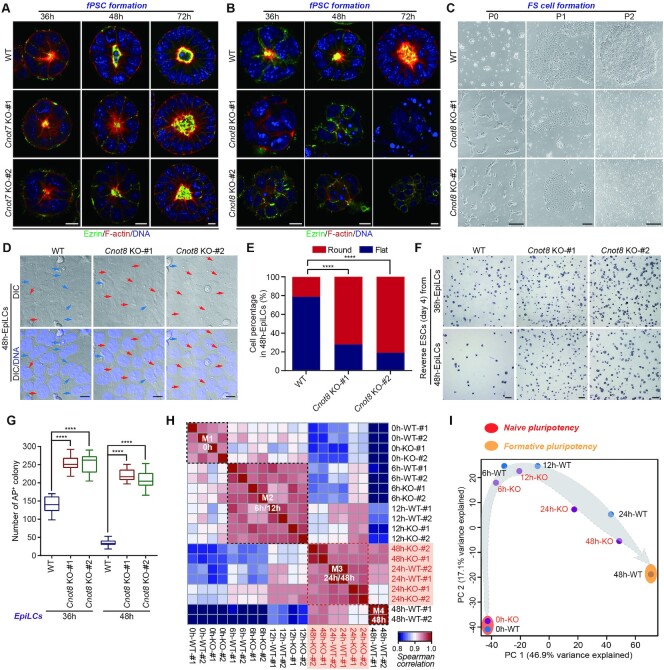
*Cnot8* is required for the differentiation of naïve ESCs into formative state. (**A**, **B**) Immunostaining analysis of WT, *Cnot7* KO, and *Cnot8* KO Epiblastoids at 36 h, 48 h, and 72 h after their differentiation. Apical lumen was labeled by Ezrin (green) and F-actin (Phalloidin, red) staining. DNA was counterstained with Hoechst 33342 (blue). Scale bar, 10 μm. (**C**) Morphology of WT and *Cnot8* KO FS cells. After induction with FS cell conditions, the cells were passaged and imaged. Scale bars, 20 μm (left panel), 50 μm (middle panel), and 20 μm (right panel). (**D**, **E**) Morphology (**D**) and quantification (**E**) of WT and *Cnot8* KO 48h-EpiLCs. DNA was counterstained with Hoechst 33342 (blue). The red and blue arrows indicated the round and flat cells, respectively. Data were represented as mean ± SEM. n = 3 biological replicates. ****ρ < 0.0001 by two-tailed Student's *t* test. Scale bar, 10 μm. (**F**, **G**) WT and *Cnot8* KO 36h- and 48h-EpiLCs were cultured in 2i/LIF conditions for 4 days and subjected to Alkaline phosphatase (AP) staining (**F**) and quantification (**G**). Data were represented as mean ± SEM. n = 3 biological replicates. ****ρ < 0.0001 by two-tailed Student's *t* test. Scale bar, 400 μm. (**H**) Hierarchical clustering based on the expression of all genes in WT and *Cnot8* KO cells at indicated time points of EpiLC differentiation. M, Module. (**I**) Principal component analysis (PCA) based on the expression of all genes for WT and *Cnot8* KO cells at specific time points of EpiLC differentiation. Red and orange regions indicated naïve and formative pluripotent states. The gray arrow indicated the differentiation direction of the cells. PC1 and PC2 represented the two dimensions of gene expressions, which accounted for 46.9% (PC1) and 17.1% (PC2) of the genes analyzed.

Since ablation of either *Cnot7* or *Cnot8* caused no substantial effects on the state of naïve pluripotency, *Cnot7* or *Cnot8* KO ESCs were differentiated into the intermediate states of PSCs between naive and primed states that were recently developed (11–14). *Cnot7* KO fPSCs were successfully obtained (Figure 2A). Cnot8 mRNA and protein was expressed in the different states of PSCs (Supplementary Figure S2G and H). Interestingly, RSCs and XPSCs could be differentiated from *Cnot8* KO ESCs (Supplementary Figure S3A and B), probably representing their earlier developmental states (11,13). However, *Cnot8* KO fPSCs and FS cells could not be established (Figure 2B and C). Then, we used another system of epiblast-like cells (EpiLCs) to investigate the role of *Cnot8* during naïve-to-formative progression (8,63). After 48 h of differentiation, more round cells were observed in *Cnot8* KO EpiLCs when compared with WT controls (Figure 2D-E and Supplementary Figure S3C), suggesting a delayed differentiation of these cells, probably consistent with the changes of cellular morphology during the exit from naïve ground state (64,65). Compared with the controls, 24h-EpiLCs differentiated from *Cnot8* KO ESCs possessed the increased round cells and displayed slightly higher AP staining (Supplementary Figure S3D-F), indicating the defects of pluripotency exit in these cells. To confirm these defects, reverse ESC (rESC) formation assay was carried out for the EpiLCs from *Cnot8* KO ESCs and WT controls at 36 h and 48 h after differentiation as previously reported (Supplementary Figure S3G) (66). Our results showed that the EpiLCs from *Cnot8* KO ESCs formed more AP-positive colonies than those from WT controls (Figure 2F and G). These results suggest that disruption of *Cnot8* impairs the exit of mouse ESCs from naïve state.

Next, we performed RNA-seq for the EpiLCs induced from WT and *Cnot8* KO ESCs at 6 h, 12 h, 24 h, and 48 h after differentiation and combined these data with the data of ESCs (0h-WT and 0h-KO). Hierarchical clustering analysis of all expressed genes showed that the 20 samples from *Cnot8* KO and WT ESCs at the 5 time points were divided into four modules (M1-M4), in which 6h- and 12h-EpiLCs from *Cnot8* KO and control ESCs were clustered into M2. M2 was separated from M1 that was composed of 0h-WT and 0h-KO ESCs (Figure 2H). Interestingly, 48h-KO, 24h-WT, and 24h-KO EpiLCs were assembled into the M3, which was separated from M4 constituted of 48h-WT EpiLCs (Figure 2H), suggesting a delayed progression of *Cnot8* KO 48h-EpiLCs. Principal component analysis (PCA) also confirmed the delayed transition from naïve to formative state of *Cnot8* KO EpiLCs (Figure 2I). The increased transcriptional noise was also observed in *Cnot8* KO ESCs (S/LIF) and 48h-EpiLCs compared with the controls (Supplementary Figure S4A-I) (59,60), indicating that *Cnot8* disruption affects the heterogeneity of ESCs and EpiLCs. Altogether, these data suggest that *Cnot8* depletion impairs the differentiation of ESCs at ∼24 h.

### Cnot8 eliminates the mRNAs of a large number of genes during the differentiation of ESCs

**Figure 3.**
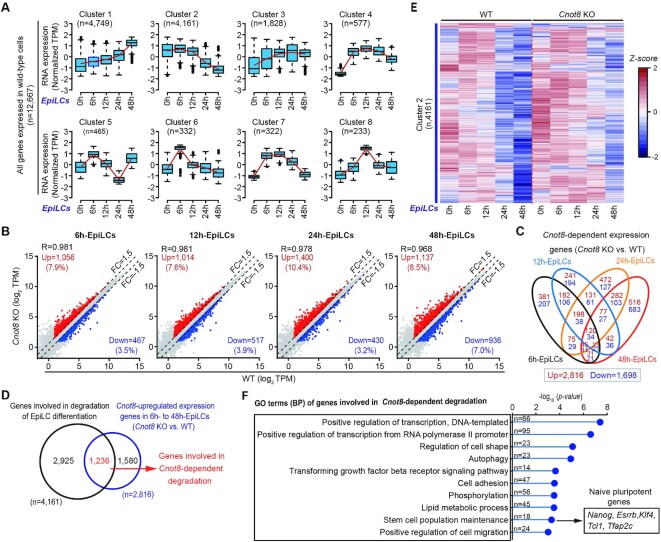
Cnot8 eliminates the mRNAs of a large number of genes during the differentiation of ESCs. (**A**) Boxplots showing clusters of genes during normal EpiLC differentiation according to the similar expression profiles. The top 8 clusters (n, 12,667) were generated using the Markov clustering algorithm. The red line indicated the mean expression level of each cluster. The middle black lines in the boxes represented the medians of expression level. The number of genes in each cluster was shown at the top of each panel. (**B**) Scatter plots showing expression profiles of the genes from 6 h to 48 h after WT and *Cnot8* KO EpiLC differentiation. The genes whose expressions were increased or decreased more than 1.5-fold in *Cnot8* KO cells compared with the normal controls at each time point were highlighted in red and blue. (**C**) Venn diagram showing intersection of differentially expressed genes (*Cnot8*-dependent expression genes) in *Cnot8* KO cells during their EpiLC differentiation. The numbers of genes with upregulated or downregulated expression were marked with red and blue. (**D**) Venn diagram showing the overlapped genes of the presumed genes (n, 4,161) involved in degradation of EpiLC differentiation and *Cnot8*-upregulated expression genes (n, 2,816). The numbers of each class genes were indicated with specific colors. (**E**) Heatmap showing expression profiles of the presumed genes in cluster 2 involved in degradation during the differentiation of WT and *Cnot8* KO ESCs. (**F**) GO term (BP) analysis of the genes (n, 1,236) involved in *Cnot8*-dependent degradation. Data were shown as -log_10_ (ρ-value). Gene number (n) of each GO term was shown in the middle. Representative naïve pluripotent genes were listed on the right.

As described above, Ccr4-Not complex is best known as a regulator of deadenylation and stability of mRNAs in different cell contexts (29). We asked whether Cnot8 sculpts the stability and subsequent decay of mRNAs during the differentiation of ESCs. We firstly compared the patterns with hidden Markov model (HMM) clustering algorithm to separate the downregulated and upregulated expression of the genes during normal EpiLC formation (67). In this analysis, ∼94.3% (12,667/13,428) genes were categorized into the top 8 clusters (Figure 3A and Supplementary Figure S5A). Among these 8 clusters, Cluster 2 accounted for ∼32.8% genes (4,161/12,667) and their expression profiles displayed a continuous pattern of downregulation, assumed as the genes involved in degradation of EpiLC differentiation (Figure 3A). We also examined the genes with upregulated expression in *Cnot8* KO EpiLCs through comparing with normal controls at specific time points (Figure 3B and Supplementary Table S4). When combining all data during EpiLC formation, we obtained 2,816 genes whose expressions were upregulated in *Cnot8* KO cells (Figure 3C). Compared with the controls, the expressions of some (n, 120) of these genes were continually upregulated, while the expressions of many other genes were changed at specific time points in *Cnot8* KO EpiLCs during the differentiation (Figure 3C).

Then, we compared the genes (n, 2,816) with upregulated expression in *Cnot8* KO cells with the genes (n, 4,161) involved in degradation of EpiLC differentiation. We totally obtained 1,236 genes whose expressions were upregulated in *Cnot8* KO cells compared with the controls. We named these genes as the genes involved in *Cnot8*-dependent degradation (Figure 3D and Supplementary Table S5). These genes accounted for ∼29.7% (1,236/4,161) of total genes involved in degradation of EpiLC differentiation (Figure 3E). Gene Ontology (GO) analysis showed that 1,236 genes involved in *Cnot8*-dependent degradation were mainly enriched in lipid metabolic process and stem cell population maintenance, including *Nanog*, *Esrrb*, *Klf4*, *Tfap2c*, and *Tcl1* (Figure 3F). Taken together, these data suggest that Cnot8 plays a crucial role in the clearance of a great number of mRNAs including those encoded by naïve TFs during the differentiation of PSCs into formative state.

### Cnot8 regulates naïve gene expression by controlling mRNA stability

**Figure 4.**
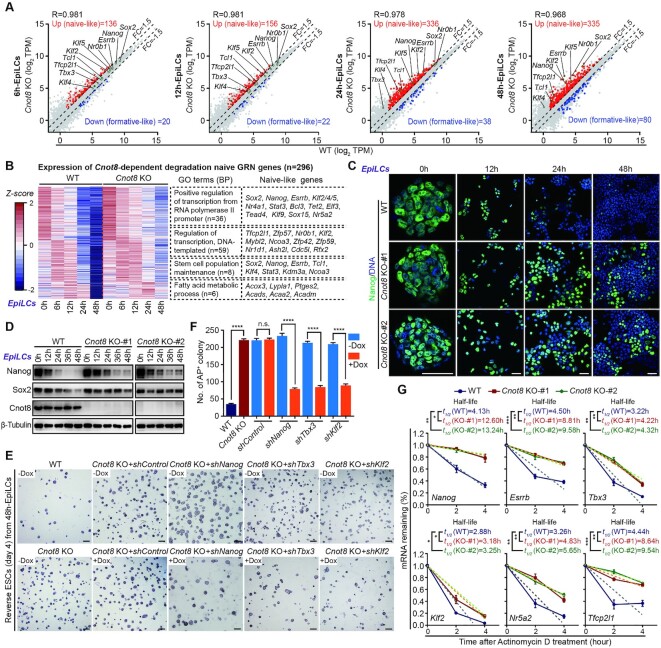
Cnot8 regulates naïve gene expression by controlling mRNA stability. (**A**) Scatter plots showing expression profiles of the genes from 6 h to 48 h after WT and *Cnot8* KO EpiLC differentiation. Genes whose expressions were increased (naïve-like genes) or decreased (formative-like genes) more than 1.5-fold in *Cnot8* KO cells compared with the controls at each time point were highlighted in red and blue, respectively. Representative general and naïve pluripotent genes were also shown in each panel according to the expression levels in WT and *Cnot8* KO cells. (**B**) Heatmap of the mRNA expression of all naïve GRN-like genes (n, 296) depending on the regulation of *Cnot8* during EpiLC differentiation. Selected enrichment GO terms were shown in the middle. Representative naïve-like genes in different terms were listed on the right. (**C**) Immunostaining results of Nanog protein (green) in WT and *Cnot8* KO cells during their EpiLC differentiation. DNA was counterstained with Hoechst 33342 (blue). Scale bar, 50 μm. (**D**) Western blot results of Nanog and Sox2 proteins in WT and *Cnot8* KO cells during their EpiLC differentiation. β-Tubulin served as a loading control. (**E**, **F**) AP staining (**E**) and quantification (**F**) of AP-positive colonies of rESCs from WT, *Cnot8* KO, and the *Cnot8* KO 48h-EpiLCs with the knockdown of *Nanog*, *Tbx3*, or *Klf2* by *shNanog*, *shTbx3*, or *shKlf2* construct. The expression of *Nanog*, *Tbx3*, and *Klf2* was downregulated in the *Cnot8* KO cells overexpressed with TetON-*shNanog*, TetON-*shTbx3*, or TetON-*shKlf2* after the treatment with (+) or without (-) Dox (2 μg/ml). Data were represented as mean ± SEM. n = 3 biological replicates. ****ρ < 0.0001, ns, not significant by two-tailed Student's *t* test. Scale bars, 300 μm. (**G**) mRNA stability analysis for representative naïve pluripotent genes. The expression levels of naïve pluripotent genes were measured by qRT-PCR following inhibition of transcription at 0, 2, or 4 h after the treatment of actinomycin D. The *t_1/2_* value reflected mRNA half-life of individual gene. The dashed lines represented linear regression of the measurement. The values at 0 h were set as 1. Data were represented as mean ± SEM. n = 3 biological replicates. *ρ < 0.05, **ρ < 0.01, ***ρ < 0.001 by two-tailed Student's *t* test.

To explore whether Cnot8 broadly modulates the clearance of mRNAs encoded by naïve-like genes, we compared the genes involved in *Cnot8*-dependent degradation with 2,684 naïve-like genes (14), and identified 539 naïve-like genes related with *Cnot8*-dependent degradation (Supplementary Figure S5B-C and Table S6). When investigating these 539 naïve-like genes at specific time points, we identified 136, 156, 336, and 335 genes in 6h-, 12h-, 24h-, and 48h-EpiLCs, respectively (Figure 4A and Supplementary Figure S5D). Strikingly, we observed many naïve TFs, such as *Nanog*, *Esrrb*, *Klf4/5*, *Tcl1*, *Tfcp2l1*, and *Nr5a2*, which are part of naïve gene regulatory networks (GRNs) (17,25), and whose expressions were especially upregulated in the *Cnot8* KO EpiLCs at 24 h and 48 h after differentiation (Figure 4A). The mRNA degradation of many naïve TFs was modulated by *Cnot8* around 24 h of EpiLC formation, suggesting a possibility that naïve GRNs should be timely dissolved and eliminated during the exit of naïve pluripotency state.

To expand naïve GRNs of PSCs, we focused on the genes with upregulated expression in 6h-, 12h-, 24h-, and 48h-*Cnot8* KO EpiLCs. Among these genes, 33 genes exhibited a pattern of continually increased expression in *Cnot8* KO EpiLCs from 6h to 48h after differentiation, and 120 genes exhibited increased expression in both 24h- and 48h-*Cnot8* KO EpiLCs, another 81 and 128 genes were only highly expressed in 24h-EpiLCs or 48h-EpiLCs, compared with the controls (Supplementary Figure S5D). Due to the defects of transition of *Cnot8* KO cells around 24 h of EpiLC formation, the genes only highly expressed in 24h-EpiLCs or 48h-EpiLCs might be also important for the naïve-to-formative transition. Thus, we combined all of these genes and totally obtained 362 genes (Supplementary Figure S5D). Then, we excluded the genes with upregulated expression in *Cnot8* KO ESCs, which might play mild role in naïve-to-formative transition. Eventually, we obtained 296 genes (Supplementary Table S7), including 45 TFs and 19 transcription cofactors according to the public database (AnimalTFDB3.0). These 296 naïve-like genes might function during naïve-to-formative state transition, thus were designated as naïve GRN-like genes. GO analysis showed that these naïve GRN-like genes were enriched in regulation of transcription, stem cell population maintenance, and metabolic pathway, including many TFs of GRNs, such as *Sox2*, *Nanog*, *Esrrb*, *Klf4/5*, *Tfcp2l1*, *Tcl1*, *Nr0b1*, as well as other pluripotent gene *Klf2*, *Nr4a1*, *Bcl3*, *Zfp42*, *Zfp57*, *Lefty2*, *Gpx2*, and *Fbxo15* (Figure 4B). qRT-PCR results showed that mRNA expression levels of the majority of these genes were gradually declined during normal ESC differentiation, but exhibited a delayed degradation in *Cnot8* KO cells especially after 18–24 h of EpiLC induction (Supplementary Figure S5E and F). The upregulated expression levels of Nanog and Sox2 proteins in *Cnot8* KO EpiLCs were confirmed by immunostaining and Western blot analyses with specific antibodies (Figure 4C-D and Supplementary Figure S5G-H). Additionally, such delayed trends of downregulated expression of naïve pluripotent genes were observed by qRT-PCR and immunostaining analyses in *Cnot8* KO cells during the progression from ESCs to RSCs or XPSCs (Supplementary Figure S6A-D).

Previous studies reported that the enforced expression or retention of pivotal naïve TF *Nanog*, *Tbx3*, *Esrrb*, *Klf2/4/5*, *Tfcp2l1*, *Nr0b1*, and *Prdm14* promotes ESC self-renewal and impairs their differentiation (68–73). To address whether the decreased expression of naïve GRN-like genes was capable of restoring the defects in the differentiation of *Cnot8* KO ESCs, we selected *Nanog*, *Klf2*, *Klf4*, and *Tbx3* to generate their stable cell lines of *Cnot8* KO ESCs with doxycycline (Dox)-inducible knockdown system using specific short hairpin RNAs (shRNAs) (54,55). We successfully obtained three stable ESC lines that specifically targeted the expression of *Nanog*, *Klf2*, or *Tbx3* (Supplementary Figure S6E). After treatment with Dox for 72 h, ∼80% of *Nanog*, *Klf2*, or *Tbx3* mRNA was knockdown in *Cnot8* KO ESCs (+Dox) compared with controls (-Dox) (Supplementary Figure S6E). Then, these *Cnot8* KO ESCs treated with Dox were induced into 48h-EpiLCs which were assayed for the clonogenicity of rESCs. Our results showed that specific knockdown of *Tbx3* in *Cnot8* KO ESCs partly reduced the formation of rESC colonies (Figure 4E and F), consistent with the roles of *Tbx3* during ESC differentiation (74,75). The knockdown of *Nanog* or *Klf2* also partly restored the forming of rESC colonies in *Cnot8*-deleted ESCs (Figure 4E and F). Immunostaining assay revealed that the degradation of Nanog protein was partially restored by the knockdown of *Tbx3* or *Klf2* in *Cnot8* KO EpiLCs at 24 h of differentiation (Supplementary Figure S6F and G).

To investigate whether *Cnot8* is involved in the decay of mRNAs, we performed mRNA stability assay for representative naïve GRN genes in *Cnot8* KO and normal ESCs as previously reported (76). After treatment with transcription inhibitor actinomycin D, qRT-PCR was used to examine the expression abundances of target mRNAs in *Cnot8* KO and normal ESCs (Supplementary Figure S6H). Our results showed that the half-life (t*_1/2_*) of the naïve mRNAs encoded by TFs, such as *Nanog*, *Esrrb*, *Tbx3*, *Klf2*, *Nr5a2*, and *Tfcp2l1*, were significantly longer in *Cnot8* KO ESCs than those in normal controls, suggesting that Cnot8 regulates the mRNA stability and decay of naïve TFs in mouse ESCs (Figure 4G and Supplementary Figure S6I). Altogether, these data suggest that Cnot8 of Ccr4-Not complex eliminates naïve GRNs through regulating their mRNA stability or decay during the exit from naïve state.

### Cnot8 globally regulates the poly(A) tail lengths of mRNAs

The stability and decay of mRNAs is associated with the lengths of poly(A) tails (77–79). Thus, we investigated the poly(A) tail sizes of mRNAs in 24h-EpiLCs using PAIso-seq, a genome-wide method to quantify the lengths of RNA poly(A) tails (58). We constructed PAIso-seq libraries for the 24h-EpiLCs from *Cnot8* KO and control ESCs as we previously described (58). With a filtering criterion of pass > 7 in all cells, 12,838 (803,537 transcripts) and 13,845 (1,323,361 transcripts) genes were captured from WT and *Cnot8* KO cells. We firstly analyzed the global distribution of poly(A) tail lengths of transcripts in the independent replicates for each genotype. Our results showed that the transcripts in the duplicates displayed a similar distribution of poly(A) tail lengths (Supplementary Figure S7A), suggesting the reliability of these PAIso-seq data.

**Figure 5.**
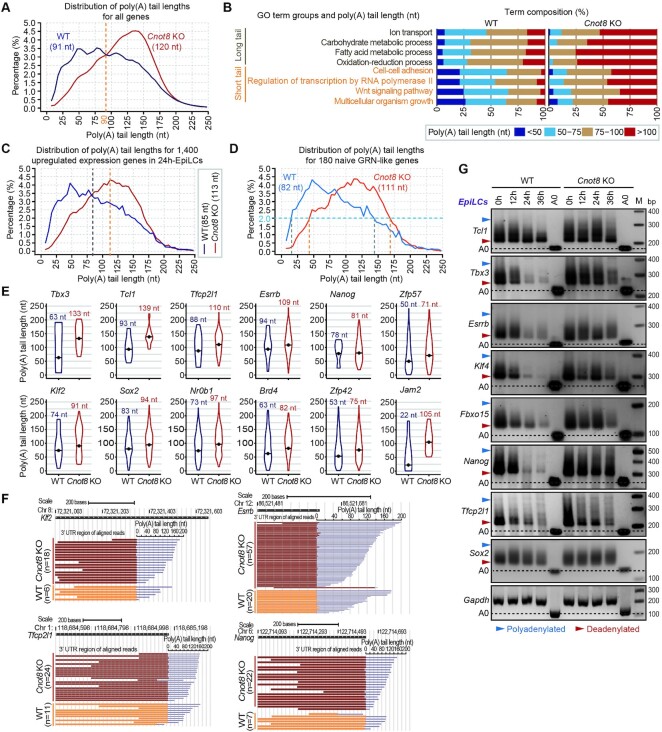
Cnot8 globally regulates the poly(A) tail lengths of mRNAs. (**A**) PAIso-seq assay showing distribution of poly(A) tail lengths for all genes in WT and *Cnot8* KO 24h-EpiLCs. The median poly(A) tail lengths for all genes of each genotype were presented in parentheses. A yellow dotted line indicated the shifted distribution of the poly(A) tail lengths for all genes in *Cnot8* KO cells. nt, nucleotide. (**B**) Functional categorization of the pairwise genes according to their geometrical mean lengths of poly(A) tails. In normal cells (WT), the genes involved in ion transport, carbohydrate metabolic process, fatty acid metabolic process, and oxidation-reduction process were categorized into the groups containing genes with long poly(A) tails (> 50% genes, poly(A) > 75 nt) in the upper panel (gray), while others with short poly(A) tails (> 50% genes, poly(A) < 75 nt) in the lower panel (orange). The full list was presented in Supplementary Table S8. (**C**) PAIso-seq assay showing distribution of poly(A) tail lengths for 1,400 upregulated expression genes in WT and *Cnot8* KO 24h-EpiLCs (related to Figure 3B). The median poly(A) tail lengths for these genes of each genotype were presented in parentheses and marked by vertical lines. (**D**) PAIso-seq assay showing distribution of poly(A) tail lengths for 180 naïve GRN-like genes in WT and *Cnot8* KO cells. The median lengths of poly(A) tails for these genes of each genotype were presented in parentheses. Three types of dotted lines with different colors (light blue, gray, and orange) indicated the shifted distribution of the poly(A) tail lengths for these genes. (**E**) Violin plots showing poly(A) tail lengths for representative pluripotent genes in WT and *Cnot8* KO cells. The geometric mean poly(A) tail length for individual gene in each sample was shown with black dots and presented at top. (**F**) Examples of PAIso-seq assay showing poly(A) tails for representative naïve TF *Klf2*, *Tfcp2l1*, *Nanog*, and *Esrrb*. Brown (*Cnot8* KO) and orange (WT) bars indicated the 3′ UTR regions that were aligned the reads to each transcript. Light blue bars were poly(A) tails of the transcripts. n, number of the transcripts. (**G**) PAT assay for representative pluripotent genes in WT and *Cnot8* KO ESCs and EpiLCs at specific time points. *Gapdh* served as an internal control. The black dashed lines represented the bands without poly(A) tail (A0). The light blue and red arrowheads indicated the polyadenylated and deadenylated bands, respectively. M, marker.

For mRNAs encoded by the nuclear genome, the fraction of transcripts with poly(A) tails of 90–200 nt was increased, while the proportion of transcripts with poly(A) tails of 5–90 nt was decreased in *Cnot8* KO cells (Figure 5A). When compared the pairwise genes (n, 6,937) that simultaneously expressed in *Cnot8* KO and control cells, the median lengths of poly(A) tails for these genes in *Cnot8* KO cells (121 nt) were apparently longer than those in control cells (92 nt) (Supplementary Figure S7B). GO term analysis suggested that these genes associated with regulatory functions, such as cell-cell adhesion, transcription regulation, Wnt signaling pathway, and multicellular organism growth, were inclined to possess relatively short poly(A) tails (> 50% genes, mRNA poly(A) < 75 nt), while the genes with constitutive functions, including ion transport, carbohydrate metabolic process, fatty acid metabolic process, and oxidation-reduction process, tended to have longer poly(A) tails (> 50% genes, mRNA poly(A) > 75 nt) in normal cells. We also noticed that the expression of mRNAs with shorter poly(A) tails might be sensitive to the depletion of *Cnot8* than those of mRNAs with longer poly(A) tails (Figure 5B and Supplementary Figure S7C-E and Table S8).

To investigate the relationship between the poly(A) tail lengths and expression levels of mRNAs, we analyzed the 1,400 genes whose expressions were upregulated in 24h-EpiLCs compared with the controls (Figure 3B). Our results showed that the poly(A) tail lengths (113 nt) for these genes in *Cnot8* KO cells were significantly longer than those in controls (85 nt) (Figure 5C). To further investigate whether *Cnot8* regulates the poly(A) tail sizes for the functional genes, we explored expression abundances and poly(A) tail lengths of mRNAs in naïve GRN-like genes whose expressions were upregulated in 24h-EpiLCs. We identified 180 naïve GRN-like genes whose transcripts possessed much longer poly(A) tails in *Cnot8* KO cells (111 nt) than those in controls (82 nt) (Figure 5D). When exploring the distribution with 2% cutoff, the poly(A) lengths of these transcripts distributed from ∼15 nt to ∼140 nt in controls, whereas spread from ∼40 nt to ∼160 nt in *Cnot8* KO cells (Figure 5D). We then analyzed the changes of poly(A) tail lengths for the specific naïve TFs of GRNs in *Cnot8* KO cells. Compared with controls, the poly(A) tails for numerous naïve TFs, such as *Tbx3*, *Klf2*, *Tfcp2l1*, *Tcl1*, *Esrrb*, *Nr0b1*, *Jam2*, and *Zfp57*, were remarkably prolonged in *Cnot8*-deficient cells (Figure 5E-F and Supplementary Figure S8A and B). Probably consistent with its increased expression levels, poly(A) tail lengths of *Cnot7* transcripts dramatically increased in *Cnot8* KO cells (Supplementary Figure S8C). We also observed that the depletion of *Cnot8* induced a conspicuous increase of poly(A) tail lengths for the genes involved in RNA degradation pathway, such as *Tob1* and *Pabpc1* (Supplementary Figure S8C). Poly(A) test (PAT) assay confirmed the observations of PAIso-seq analysis (Figure 5G and Supplementary Figure S8D). This analysis also revealed that the poly(A) tail lengths for these genes were progressively shortened during the EpiLC differentiation of control ESCs, whereas the shortening process was apparently impaired in *Cnot8*-deleted EpiLCs, especially after 24h of ESC differentiation (Figure 5G and Supplementary Figure S8D). Taken together, these results suggest that Cnot8 post-transcriptionally modulates the stability or clearance of GRN mRNAs through regulating their poly(A) tail lengths.

### Cnot8 regulates mRNA poly(A) tail lengths through its deadenylase activity and Ccr4-Not complex

**Figure 6.**
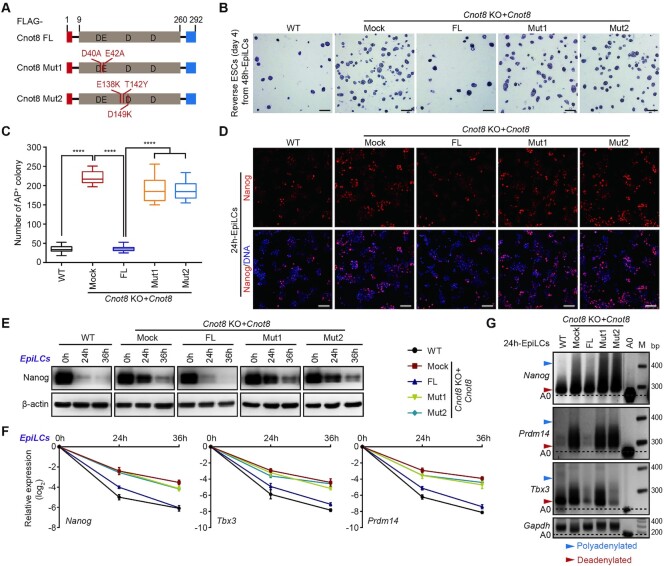
Cnot8 regulates mRNA poly(A) tail lengths through its deadenylase activity and Ccr4-Not complex. (**A**) Schematic representation of Cnot8 full-length (FL) protein and various mutant fragments. The mutant positions of amino acids were indicated. (**B**, **C**) 48h-EpiLCs induced from WT and the rescued ESCs were cultured in 2i/LIF medium for 4 days, and subjected to AP staining (**B**) and quantification (**C**) of AP-positive colonies. Data were represented as mean ± SEM. n = 3 biological replicates. ****ρ < 0.0001 by two-tailed Student's *t* test. *Cnot8* KO ESCs overexpressed with empty vector (Mock), Cnot8 FL, Cnot8 Mut1, and Cnot8 Mut2 were regarded as the rescued ESCs. Scale bar, 300 μm. (**D**) Immunostaining results of Nanog protein (red) in WT and the rescued cells at 24 h after EpiLC differentiation. DNA was counterstained with Hoechst 33342 (blue). Scale bar, 100 μm. (**E**) Western blot results of Nanog in WT and rescued cells at specific time points of EpiLC differentiation. β-actin was used as a loading control. (**F**) qRT-PCR analysis of the expression of representative naïve pluripotent genes in WT and the rescued cells at specific time points after EpiLC differentiation. The values in ESCs were set as 0. Data were represented as mean ± SEM. n = 3 biological replicates. (**G**) PAT assay for representative naïve pluripotent genes in WT and the rescued cells at 24 h after EpiLC differentiation. *Gapdh* was used as an internal control. The black dashed lines represented the bands without poly(A) tail (A0). The light blue and red arrowheads indicated the polyadenylated and deadenylated bands, respectively. M, marker.

The deadenylase activity of Cnot8 depends on two key residues D40 and E42 coordinating magnesium in the conserved DEDD domain *in vitro* (45). Thus, we engineered double substitutions at D40A and E42A of FLAG-tagged Cnot8 (Mut1) to block its deadenylase activity (Figure 6A and Supplementary Figure S9A). We expressed FLAG-tagged Cnot8 FL (normal Cnot8 full length) or Mut1 in *Cnot8* KO ESCs with the delivering system of lentivirus (Supplementary Figure S9B). rESC formation assay showed that the enforced expression of Cnot8 FL but not Mut1 rescued the delayed differentiation phenotype of *Cnot8* KO ESCs (Figure 6B and C). Immunostaining and Western blot analyses revealed that Cnot8 FL but not Mut1 rescued the dysregulated expression of Nanog protein in *Cnot8* KO cells during EpiLC differentiation (Figure 6D-E and Supplementary Figure S9C). qRT-PCR analysis showed that Cnot8 FL but not Mut1 restored the suppressed mRNA decay of *Nanog*, *Tbx3*, and *Prdm14* in *Cnot8* KO cells (Figure 6F). PAT assay further revealed that Cnot8 normal FL rather than Mut1 was capable of restoring the delayed deadenylation for *Nanog*, *Tbx3*, and *Prdm14* in *Cnot8* KO cells (Figure 6G). Additionally, mRNA stability assay also confirmed that Cnot8 FL but not Mut1 restored the dysregulated half-life of naïve pluripotent mRNAs (Supplementary Figure S9D). These results reveal that the deadenylase activity of Cnot8 is critical for mRNA deadenylation and stability of naïve GRN mRNAs.

To investigate whether Cnot8 is involved in Ccr4-Not complex, we conducted coimmunoprecipitation (Co-IP) assay. Our results showed that FLAG-Cnot8 precipitated the scaffold protein Cnot1, but not Cnot7 in the lysate of mouse ESCs with anti-FLAG antibody, suggesting its physiological interaction with Cnot1 in ESCs (Supplementary Figure S9E), probably consistent with the observations that Cnot8 or Cnot7 is in different Ccr4-Not complex of HEK293FT cells (80). Previous studies show that Cnot7 directly interacts with Cnot1 via three residues of E138, T142, and E149 in its DEDD domain (Supplementary Figure S9A) (81,82), we hypothesized that Cnot8 interacted with Cnot1 through these three residues. To test this, we generated mutations for the Cnot8 at E138K, T142Y, and E149K (Mut2) (Figure 6A). Our results showed that Cnot8 FL but not Mut2 efficiently precipitated Cnot1, suggesting the important role of these three residues in their interaction (Supplementary Figure S9F). Then, we expressed FLAG-tagged Cnot8 FL or Mut2 in *Cnot8* KO ESCs to examine the function of their interaction. Our results showed that Cnot8 FL but not Mut2 rescued the dysregulated differentiation phenotypes of *Cnot8* KO ESCs, suggesting that Cnot8 functions through its interaction with Cnot1 in Ccr4-Not complex (Figure 6B-G and Supplementary Figure S9B-D). Notably, the reintroduction of Cnot7 FL into *Cnot8* KO ESCs failed to rescue the mRNA degradation during *Cnot8* KO ESC differentiation (Supplementary Figure S9G and H). Additionally, we obtained stable ESC lines that specifically targeted the expression of *Cnot1* (Supplementary Figure S10A). Knockdown of *Cnot1* in ESCs led to their differentiation and the downregulated expressions of *Oct4* and *Tfcp2l1*, as well as the upregulated expressions of trophoblast marker *Cdx2* and *Gata3* in these cells (Supplementary Figure S10B and C), consistent with the previous report (34). The protein expression levels of Oct4 and deadenylase Cnot7 and Cnot8 were decreased in *Cnot1* knockdown cells (Supplementary Figure S10D). *Cnot1* deficiency also prohibited the ESC differentiation into RSCs (Supplementary Figure S10E and F). Taken together, these results suggest that Cnot8 regulates the poly(A) lengths and stability of naïve GRN mRNAs through its deadenylase activity and Ccr4-Not complex.

### Cnot8 interacts with Tob1 and Pabpc1 to modulate mRNA clearance

Cnot8, resembling its paralog Cnot7, is not an RNA binding protein (45,83,84). RNA binding proteins might be involved in the function of Cnot8 in mRNA deadenylation and clearance of mouse PSCs. Previous reports show that CAF1 catalytic subunit of the Ccr4-Not complex is associated with cytoplasmic poly(A)-binding protein 1 (Pabpc1) through Tob1, an adapter protein (83,85–88). *Tob1* has also been showed to participate in modulating the cell propagation of ESCs by degrading *Id3* mRNA, a target gene of Bmp4 signaling (89). Therefore, we asked whether the role of Cnot8 in deadenylation and degradation of mRNAs is related with Pabpc1 and Tob1 in mouse PSCs.

**Figure 7.**
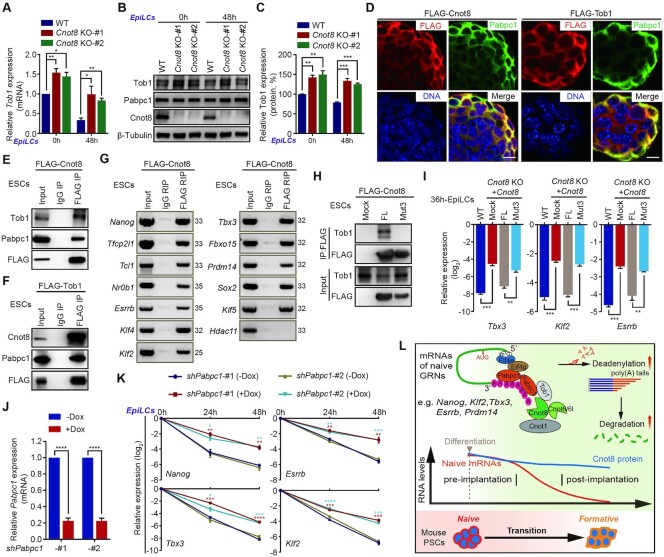
Cnot8 interacts with Tob1 and Pabpc1 to modulate mRNA clearance. (**A**) qRT-PCR analysis of *Tob1* mRNA in WT and *Cnot8* KO ESCs and 48h-EpiLCs. Data were represented as mean ± SEM. n = 3 biological replicates. *ρ < 0.05, **ρ < 0.01 by two-tailed Student's *t* test. (**B**) Western blot results of Tob1 and Pabpc1 proteins in WT and *Cnot8* KO ESCs and 48h-EpiLCs. β-actin served as a loading control. (**C**) Quantification of Tob1 protein in WT and *Cnot8* KO ESCs and 48h-EpiLCs. The levels of protein expression in ESCs were set as 100%. Data were represented as mean ± SEM. n = 3 biological replicates. **ρ < 0.01, ***ρ < 0.001 by two-tailed Student's *t* test. (**D**) Immunostaining results of Cnot8, Tob1, and Pabpc1 in ESCs. Cnot8 and Tob1 were stained with anti-FLAG antibody and Pabpc1 was stained with specific anti-Pabpc1 antibody. DNA was counterstained with Hoechst 33342. Scale bar, 10 μm. (**E**, **F**) Western blot analysis for the products of Co-IP (**E**) and reverse Co-IP (**F**) assay using anti-FLAG antibody in ESCs. IgG served as a negative control. (**G**) RIP assay showed that Cnot8 interacted with the mRNAs of representative general and naïve TFs in ESCs. mRNA abundances of co-immunoprecipitated products with anti-FLAG antibody were determined by RT-PCR with specific primers. IgG and histone *Hdac11* were used as the negative controls. The numbers of PCR cycles were presented on the right. (**H**) Co-IP assay of the interaction between FLAG-tagged Cnot8 Mut3 and Tob1 in mouse ESCs. Mock, ESCs transfected with empty vector. (**I**) qRT-PCR analysis of the expression of representative naïve pluripotent genes at 0 h and 36 h after WT and rescued ESC differentiation. The values in ESCs were set as 0. Data were represented as mean ± SEM. n = 3 biological replicates. **ρ < 0.01, ***ρ < 0.001 by two-tailed Student's *t* test. Rescued ESCs, *Cnot8* KO ESCs overexpressed with empty vector (Mock), Cnot8 FL, and Cnot8 Mut3. (**J**) qRT-PCR results showing efficiency of *Pabpc1* interference after WT ESCs ectopically expressed with two TetON-*shPabpc1* were treated with (+) or without (-) Dox (2 μg/ml) for 72 h. Data were represented as mean ± SEM. n = 3 biological replicates. ****ρ < 0.0001 by two-tailed Student's *t* test. (**K**) qRT-PCR analysis for representative naïve pluripotent genes in the cells with or without *Pabpc1* interference at specific time points of EpiLC differentiation. The values of ESCs were set as 0. Data were represented as mean ± SEM. n = 3 biological replicates. *ρ < 0.05, **ρ < 0.01, ***ρ < 0.001, ****ρ < 0.0001 by two-tailed Student's *t* test. (**L**) A schematic model of mRNA degradation modulated by Cnot8, a deadenylase of Ccr4-Not complex, during naïve-to-formative pluripotent transition. Cnot8 promotes mRNA deadenylation and degradation of naïve GRN genes at post-transcriptional level through Pabpc1/Tob1, and functions during the formative progression from naïve stem cell state.

The mRNAs and proteins of *Tob1* and *Pabpc1* were enriched in mouse ESCs, and the expression levels of *Tob1* mRNA and protein were downregulated in 48h-EpiLCs (Figure 7A-C). Probably consistent with its prolonged poly(A) tail lengths (Supplementary Figure S8C), the expression levels of *Tob1* were significantly upregulated in *Cnot8* KO cells (Figure 7A-C and Supplementary Figure S10G-H). Immunostaining assay showed that Cnot8, Tob1, and Pabpc1 were colocalized in the cytoplasm of ESCs (Figure 7D). Co-IP results of FLAG-Cnot8 showed that Tob1 and Pabpc1 were pulled down by Cnot8 (Figure 7E), and reverse Co-IP experiments of FLAG-Tob1 showed that Cnot8 and Pabpc1 were pulled down by Tob1 (Figure 7F), suggesting that Cnot8 is physically associated with Pabpc1 and Tob1 in mouse ESCs. We also observed that their interactions were not interfered by RNase A treatment and Cnot7 could pull down Tob1 and Pabpc1 in mouse ESCs (Supplementary Figure S10I and J). Next, we sought to investigate whether the complex of Cnot8/Tob1/Pabpc1 interacts with the mRNAs of selected genes of naïve GRNs and modulates their degradation. We overexpressed FLAG-Cnot8 in mouse ESCs using lentivirus and performed RNA immunoprecipitation (RIP) assay with anti-FLAG antibody. Our results showed that the mRNAs encoded by naïve TF *Nanog*, *Klf2*, *Tbx3*, *Tcl1*, *Esrrb*, *Klf4*, and *Prdm14* were enriched in the products precipitated by FLAG antibody but not normal IgG, suggesting that the Cnot8 interacts with GRN mRNAs (Figure 7G and Supplementary Figure S10K). Previous studies report that the E247A and Y260A mutations in mouse Cnot7 abolish its interaction with Tob1 (83,87), and these residues are conserved in Cnot8 (Supplementary Figure S9A). We speculated that these two residues of Cnot8 were important for its interaction with Tob1/Papbc1 and the function of mRNA degradation in mouse PSCs. Then we overexpressed FLAG-tagged Cnot8 with 247A/260A mutations (Mut3) in *Cnot8* KO ESCs (Supplementary Figure S10L and M). Co-IP assay showed that the Cnot8 Mut3 abolished the Cnot8-Tob1 interaction in mouse ESCs (Figure 7H). The overexpression of Cnot8 FL, but not Mut3 rescued the defects in the degradation of *Tbx3*, *Klf2*, and *Esrrb* mRNAs in *Cnot8* KO cells during EpiLC differentiation (Figure 7I). These results suggest that Cnot8 functions in mRNA degradation of naïve GRNs through Tob1 and Pabpc1 in mouse PSCs.

To investigate the role of Cnot8/Tob1/Pabpc1 in mouse PSCs, we further examine whether *Pabpc1* is implicated in mRNA degradation of naïve GRNs. To this end, we generated two stable ESC lines of doxycycline (Dox)-inducible knockdown cells using different short hairpin RNAs (shRNAs). After treatment with Dox for 72 h in 2i/LIF medium, ∼80% of *Pabpc1* mRNA was decreased in these ESCs (Figure 7J). The knockdown of *Pabpc1* did not interfere the expression abundances of naïve TF mRNAs in mouse ESCs (Supplementary Figure S10N), suggesting a strong regulation machinery for the expression of naïve TFs in mouse ESCs. However, the knockdown of *Pabpc1* impaired the degradation of naïve TF mRNAs during the induction of EpiLCs (Figure 7K). Taken together, these results indicate that Cnot8 of Ccr4-Not complex interacts with Tob1 and Pabpc1 to modulate the poly(A) tail lengths of naïve GRNs, thus controlling their stability and degradation during the formative pluripotent transition from naïve state (Figure 7L).

## DISCUSSION

Following implantation of embryos into the uterus, the pluripotent epiblast undergoes a series of events accompanying with the drastic transformations of morphology, metabolism, gene expression, and epigenetic states (90–92). Due to the inaccessibility of embryos at peri-implantation stage, the stabilized PSCs from mammalian early embryos provide alternative *in vitro* approaches to investigate the regulation of peri-implantation epiblast development (7,15,93). A large number of evidence suggests that *Oct4*, *Sox2*, *Nanog*, *Klf2/4/5*, *Tfcp2l1*, and *Tbx3* play important roles in mouse early embryonic development (94). These transcription factors (TFs) may form the GRNs to maintain the self-renewal and block the differentiation of naïve ESCs (25,95). During early embryonic development, the expression levels of GRN TFs are high in mouse pre-implantation embryos, while are low in post-implantation epiblast cells (96). These observations suggest that naive GRNs control the identity of PSCs and should be degraded or downregulated during the transition from naïve to formative pluripotency. Currently, how and when the GRNs are degraded or dissolved during this process remains largely unknown.

At present study, we find that Cnot8, but not other deadenylases of Ccr4-Not complex, is essential for mouse embryonic development, consistent with the previous report (62). Although *Cnot8* may play broader roles during mouse early embryonic development, *Cnot8* is critical for the transition of ESCs from naïve into formative state. Consistent with the role of Cnot8 as a deadenylase of Ccr4-Not complex, depletion of *Cnot8* resulted in the increased Poly(A) tail lengths of global transcripts, thus stabilizing or increasing the expression of a great many genes including about three hundred of naïve GRN-like genes, such as *Nanog*, *Tbx3*, and *Klf2* of GRNs, consistent with the prolonged half-life of these mRNAs. The decreased expression of *Nanog*, *Tbx3*, or *Klf2* of GRNs by shRNA partially restores the phenotypes of the delayed or impaired transition in *Cnot8* KO cells. Cnot8 full-length (FL) but not Mut1 (carrying the mutations with no enzymatic activity), Mut2 (blocking their binding sites with Cnot1), or Mut3 (abolishing the interacting sites with Tob1) can rescue the effects on their mRNA expression and deadenylation of the target mRNAs. Consistent with these observations in PSCs, *Cnot8* is required for the downregulated expression of general and naïve TF *Sox2*, *Nanog*, *Tbx3*, and *Esrrb* in pluripotent epiblast cells of *in vitro* cultured embryos. Altogether, these observations suggest that Cnot8 deadenylates mRNA poly(A) tails and reduces their stability through Ccr4-Not complex and the interaction with Tob1/Pabpc1, thus clearing naïve GRNs, and plays important roles in the formative progression from naïve state of PSCs and mouse early embryonic development.

The naïve TFs upregulated in *Cnot8* KO 24h- to 48h-EpiLCs prompt us to identify about three hundred of naïve GRN-like genes that include 59 genes involved in regulation of transcription, 8 genes associated with stem cell population maintenance, and 6 genes related with metabolism. The naïve pluripotency features of PSCs may be maintained by the expression of naïve GRN-like genes. During naïve-to-formative conversion, the expressions of these naïve GRN-like genes should be timely dissolved or cleared. For instance, the degradation defects of *Nanog*, *Tbx3*, and/or *Klf2* in *Cnot8* KO ESCs may delay their transition from naïve to formative pluripotency, and the knockdown of *Nanog*, *Tbx3*, or *Klf2* can partially rescue their delayed development in these cells. In other words, the pluripotent stem cells should be regarded as ‘naïve’ ESCs that possess high expression abundances of naïve GRN-like genes. For example, conventional ESCs cultured in 2i/LIF medium is a typical type of these ‘naïve’ ESCs. However, compared with conventional ESCs, the ESCs in 3i conditions that contain inhibitor CHIR99021, PD184352, and SU5402 may be more ‘naïve’ state (18,90). Thus, other types of naïve-like ESCs are also probably present. For instance, embryonal carcinoma (EC) cells (97), ESCs cultured in serum (2) or serum/LIF (98), iPSCs (99), CiPSCs (100), RSCs (11), and XPSCs (13) express high levels of naïve GRN-like genes and might also be regarded as ‘naïve’ ESCs. Further investigations of the roles for these naïve GRN-like genes will provide important insights into the pluripotency features of ESCs. Altogether, the generation and maintenance of these ‘naïve’ ESCs probably supports a hypothesis that ‘naïve’ pluripotency may be a phase, similar to the formative pluripotency (93).

Cnot8 and Cnot7 are Caf1 orthologs of mammalian Ccr4-Not complex and possess ∼81% identity of amino acids in their DEDD domains of deadenylases. Both deadenylases have been shown to be involved in the deadenylation and mRNA stability through Ccr4-Not complex in numerous processes. In mouse ESCs, Cnot8 or Cnot7 is separated into the different Ccr4-Not complex and interacts with the adapter protein Tob1, probably consistent with that in HEK293FT cells (80,83). Cnot8 can compensate for the role of Cnot7 in mouse ESCs and early embryonic development. Although the extremely high similarity of Cnot7 and Cnot8 in their DEDD domains of deadenylases (101), the defects of cell differentiation and mRNA decay in *Cnot8* KO cells cannot be reversed by the reintroduction of *Cnot7*, suggesting that Cnot8 specifically functions during the exit from naive state of PSCs through degrading the mRNA of naive genes. Although Cnot8 regulates mRNA deadenylation of naive genes, it also controls the deadenylation of many other mRNAs during the differentiation of ESCs. Thus, other mechanisms may control the expression of genes in the cells (79). How Cnot8 specifically functions and how the deadenylation is related with the expression level of targets in PSCs are deserved for further investigations.

In summary, our results suggest that Cnot8 promotes mRNA deadenylation and degradation of naïve regulation networks through Ccr4-Not complex and associating with Tob1 and Pabpc1, and is essential for the transition of pluripotent stem cells from naïve to formative state (Figure 7L). Therefore, our study unveils a post-transcriptional deadenylation mechanism to sculpt the fate transition of pluripotent stem cells. This study not only expands the naïve regulation networks and features of pluripotent stem cells, but provides new insights into epiblast development of early embryos in mammals.

## DATA AVAILABILITY

The RNA-seq, scRNA-seq, and PAIso-seq data generated in this study have been deposited to GEO database under total accession number GEO: GSE174425.

## Supplementary Material

gkac236_Supplemental_Files
