# Supplementary material for: Cnot8 eliminates naïve regulation networks and is essential for naïve-to-formative pluripotency transition
Source: Nucleic Acids Res. 2022 Apr 7;50(8):4414–35. doi: 10.1093/nar/gkac236 (PMC9071485; doi:10.1093/nar/gkac236)
Supplement: gkac236_Supplemental_Files [file gkac236_supplemental_files.zip › Supplementary information-proof-22.4.5.pdf]

Supplementary Information

Cnot8 Eliminates Naïve Regulation Networks and Is Essential for Naïve-to-Formative Pluripotency Transition

Yujun Quan[1,3,#], Meijiao Wang[2,#], Chengpeng Xu[1,3], Xiaoxiao Wang[1], Yu Wu[1,3], Dandan Qin[1], Yuxuan Lin[1], Xukun Lu[1], Falong Lu[2,3,*] and Lei Li[1,3,*]

[1]State Key Laboratory of Stem Cell and Reproductive Biology, Institute of Stem Cell and Regeneration, Beijing Institute of Stem Cell and Regenerative Medicine, Institute of Zoology, Chinese Academy of Sciences, Beijing 100101, China, [2]State Key Laboratory of Molecular Developmental Biology, Institute of Genetics and Developmental Biology, Innovative Academy of Seed Design, Chinese Academy of Sciences, Beijing, 100101, China, [3]University of Chinese Academy of Sciences, Beijing 100049, China

***Corresponding author**

fllu@genetics.ac.cn (F.L.L.)

lil@ioz.ac.cn (L.L.)

This file includes:

Supplementary Figure S1-S10

Supplementary Table S1-S8

Supplementary Figures

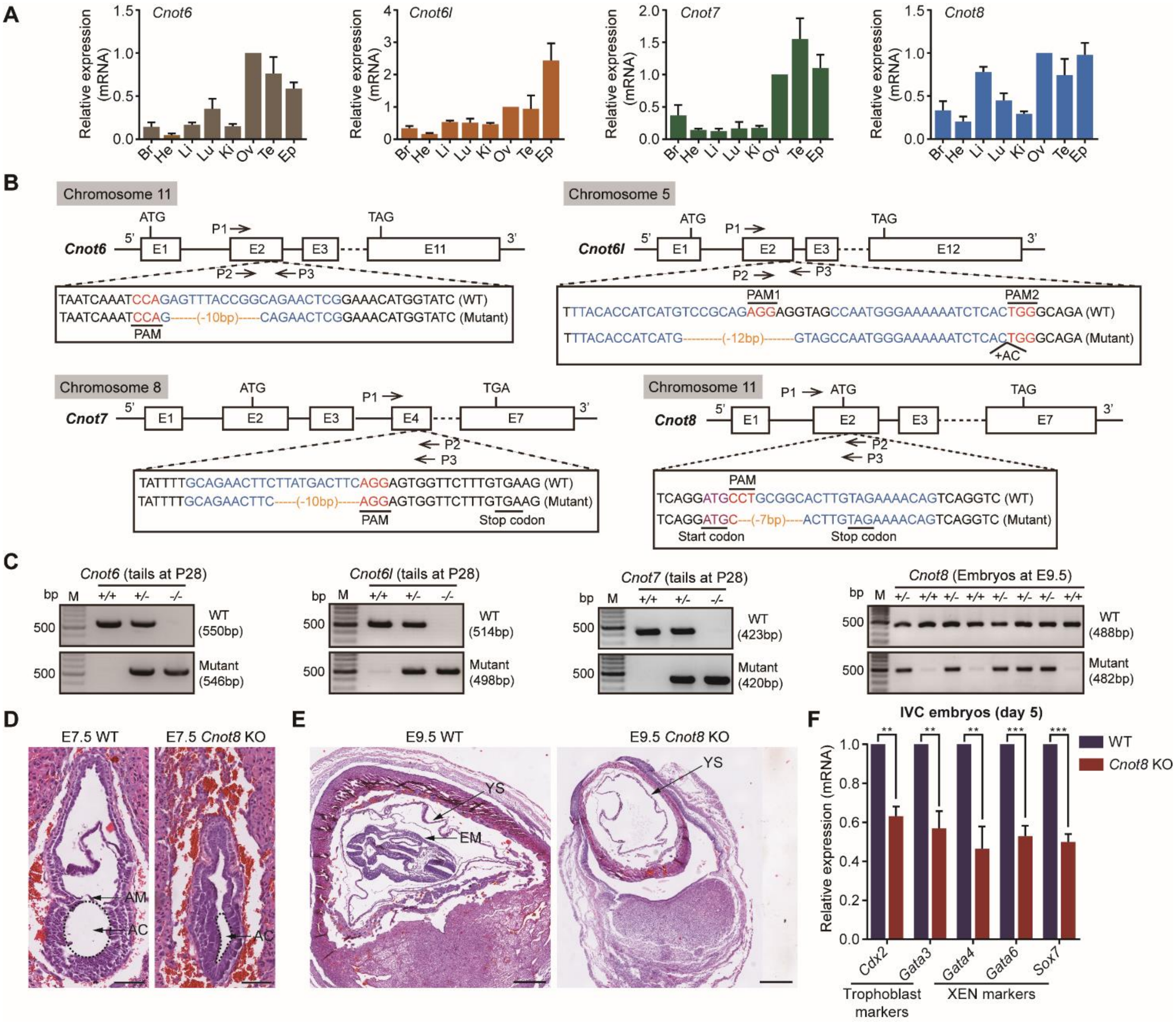

Supplementary Figure S1. *Cnot8* is essential for mouse early embryonic development.

(**A**) Quantitative RT-PCR (qRT-PCR) analysis of *Cnot6*, *Cnot6l*, *Cnot7*, or *Cnot8* mRNA in mouse different tissues. Br, Brain; He, Heart; Li, Liver; Lu, Lung; Ki, Kidney; Ov, Ovary; Te, Testis; Ep, Epididymis. Data were represented as mean ± SEM, n = 3 biological replicates. (**B**) Gene-targeting strategies of *Cnot6*, *Cnot6l*, *Cnot7*, or *Cnot8* with CRISPR/Cas9 system at specific locus of these genes. E, exon; P, primer for genotyping. (**C**) PCR results of mouse and embryo genotyping using tail (at P28) or embryonic (at E9.5) genomic DNA as template with specific primers. For *Cnot6*, the WT allele ("+") and 10-nucleotide-deletion allele ("-") were amplified with primer *Cnot6*-P1, *Cnot6*-P2, and *Cnot6*-P3. For *Cnot6l*, the WT allele ("+") and 10 (-12+2)-nucleotide-deletion allele ("-") were amplified with primer *Cnot6l*-P1, *Cnot6l*-P2, and *Cno6l*-P3. For *Cnot7*, the WT allele ("+") and 10-nucleotide-deletion allele ("-") were amplified with the primers of *Cnot7*-P1, *Cnot7*-P2, and *Cnot7*-P3. For *Cnot8*, the WT allele ("+") and 7-nucleotide-deletion allele ("-") were amplified with primer *Cnot8*-P1, *Cnot8*-P2, and *Cnot8*-P3. Primer sequences used were listed in Table S1. (**D**, **E**) WT and *Cnot8* KO

embryos derived at E7.5 (**D**) and E9.5 (**E**) were fixed and sliced for paraffin sections. The paraffin sections were subjected to H&E staining. AM, amnion; AC, amniotic cavity; YS, yolk sac; EM, embryo. Black dashed lines indicated the AC at E7.5. Scale bar, 100 μm (E7.5) and 500 μm (E9.5). (**F**) qRT-PCR analysis of trophoblast and extraembryonic endoderm (XEN) markers in WT and *Cnot8* KO IVC embryos. Data were represented as mean ± SEM, n = 3 biological replicates. **$\rho < 0.01$, ***$\rho < 0.001$ by two-tailed Student's *t* test. IVC, *in vitro* culture.

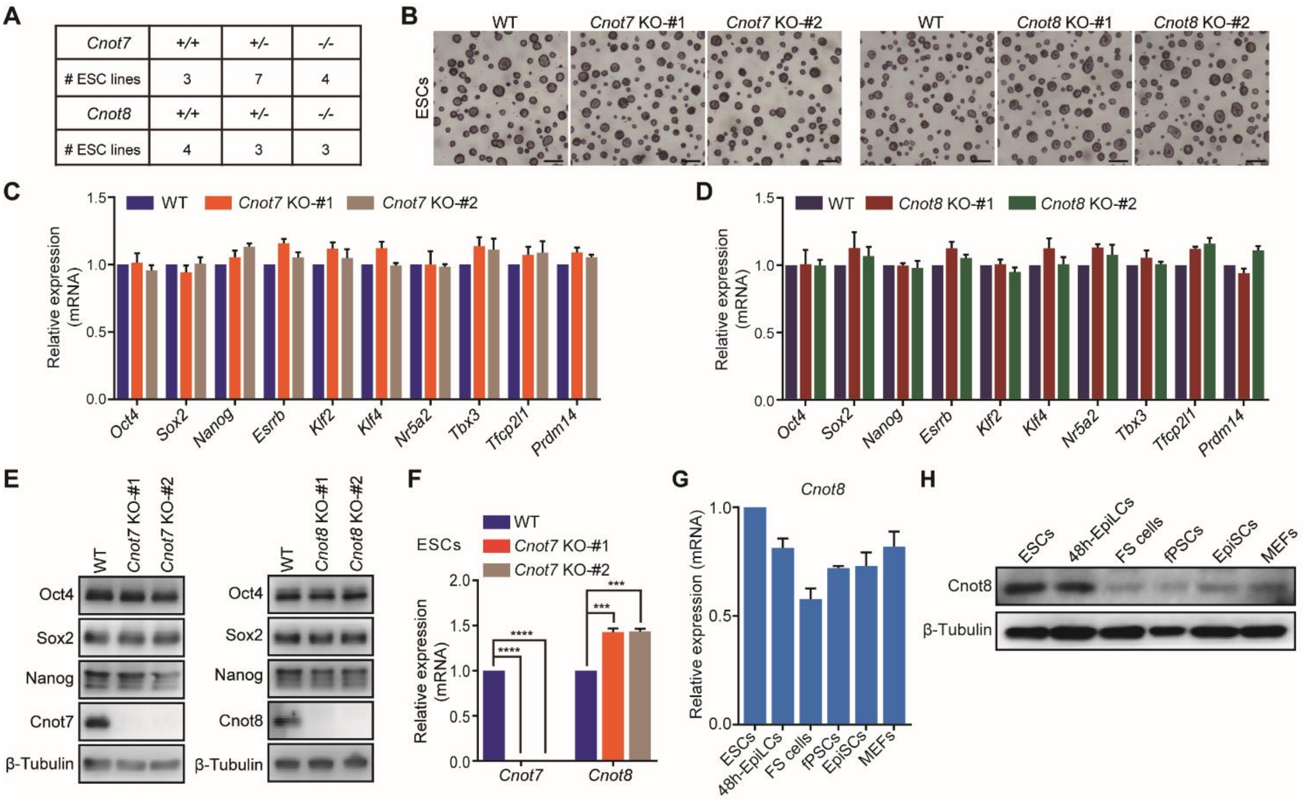

Supplementary Figure S2. Derivation and maintenance of *Cnot7* or *Cnot8* KO mESCs

(**A**) Numbers of ESC lines derived from the embryos that were collected from the natural mating of *Cnot7* or *Cnot8* heterozygous males and females. (**B**) Alkaline phosphatase (AP) staining of WT, *Cnot7* KO, and *Cnot8* KO ESCs. Scale bar, 200 μm. (**C**, **D**) qRT-PCR analysis of representative core and naïve genes in WT, *Cnot7* KO (**C**), and *Cnot8* KO (**D**) ESCs with specific primers. Data were represented as mean ± SEM. n = 3 biological replicates. (**E**) Western blot results of key developmental regulators in WT, *Cnot7* KO, and *Cnot8* KO ESCs. β-Tubulin served as a loading control. (**F**) qRT-PCR analysis of *Cnot8* mRNA in WT and *Cnot7* KO ESCs. Data were represented as mean ± SEM, n = 3 biological replicates. ***$\rho < 0.001$, ****$\rho < 0.0001$ by two-tailed Student's *t* test. (**G**) qRT-PCR analysis of *Cnot8* mRNA in normal ESCs, 48h-EpiLCs, FS cells, fPSCs, EpiSCs, and MEFs. Data were represented as mean ± SEM, n = 3 biological replicates. (**H**) Western blot results of Cnot8 protein in normal ESCs, 48h-EpiLCs, FS cells, fPSCs, EpiSCs, and MEFs. β-Tubulin served as a loading control.

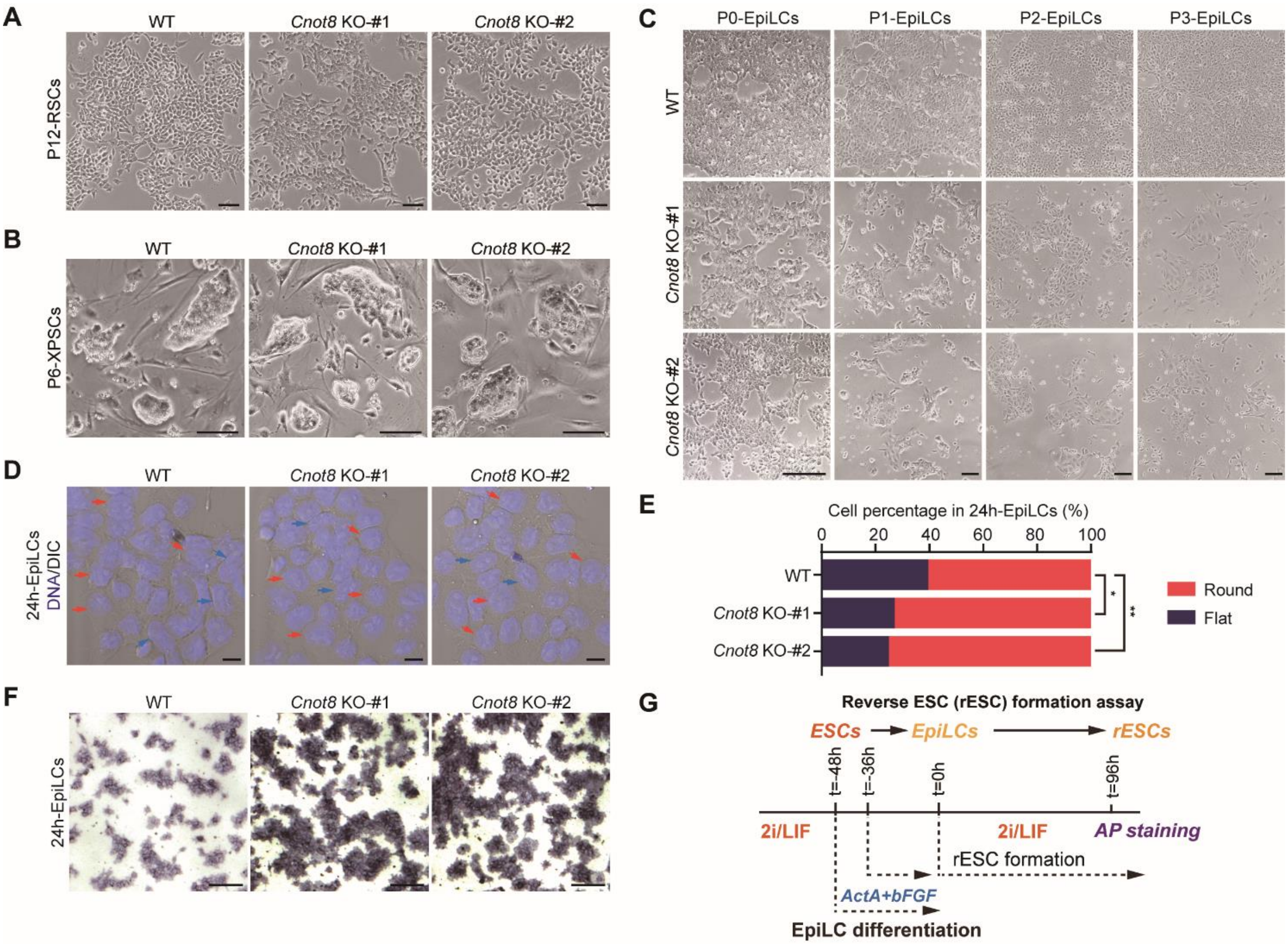

Supplementary Figure S3. *Cnot8* is required for the differentiation of naïve ESCs into formative state. (**A**, **B**) Morphology of WT and *Cnot8* KO RSCs (**A**) and XPSCs (**B**). Scale bar, 50 μm. (**C**) Morphology of WT and *Cnot8* KO EpiLCs. ESCs were induced for 48h and passaged under EpiLC formation medium supplemented with 5 μM XAV939. Scale bar, 50 μm. (**D**, **E**) Morphology (**D**) and quantification (**E**) analysis of WT and *Cnot8* KO 24h-EpiLCs. DNA was counterstained with Hoechst 33342 (blue). The red and blue arrows indicated the round and flat cells, respectively. Data were represented as mean ± SEM. n = 3 biological replicates. *$\rho < 0.05$, **$\rho < 0.01$ by two-tailed Student's *t* test. Scale bar, 10 μm. (**F**) AP staining of WT and *Cnot8* KO 24h-EpiLCs. Scale bar, 200 μm. (**G**) A schematic illustration of reverse ESC (rESC) formation assay. ESCs were cultured in EpiLC formation medium for 36 h and 48 h. These EpiLCs were dissociated into single cells, re-plated into 2i/LIF medium and cultured for 4 days in ESC medium. The rESCs were subjected to AP staining.

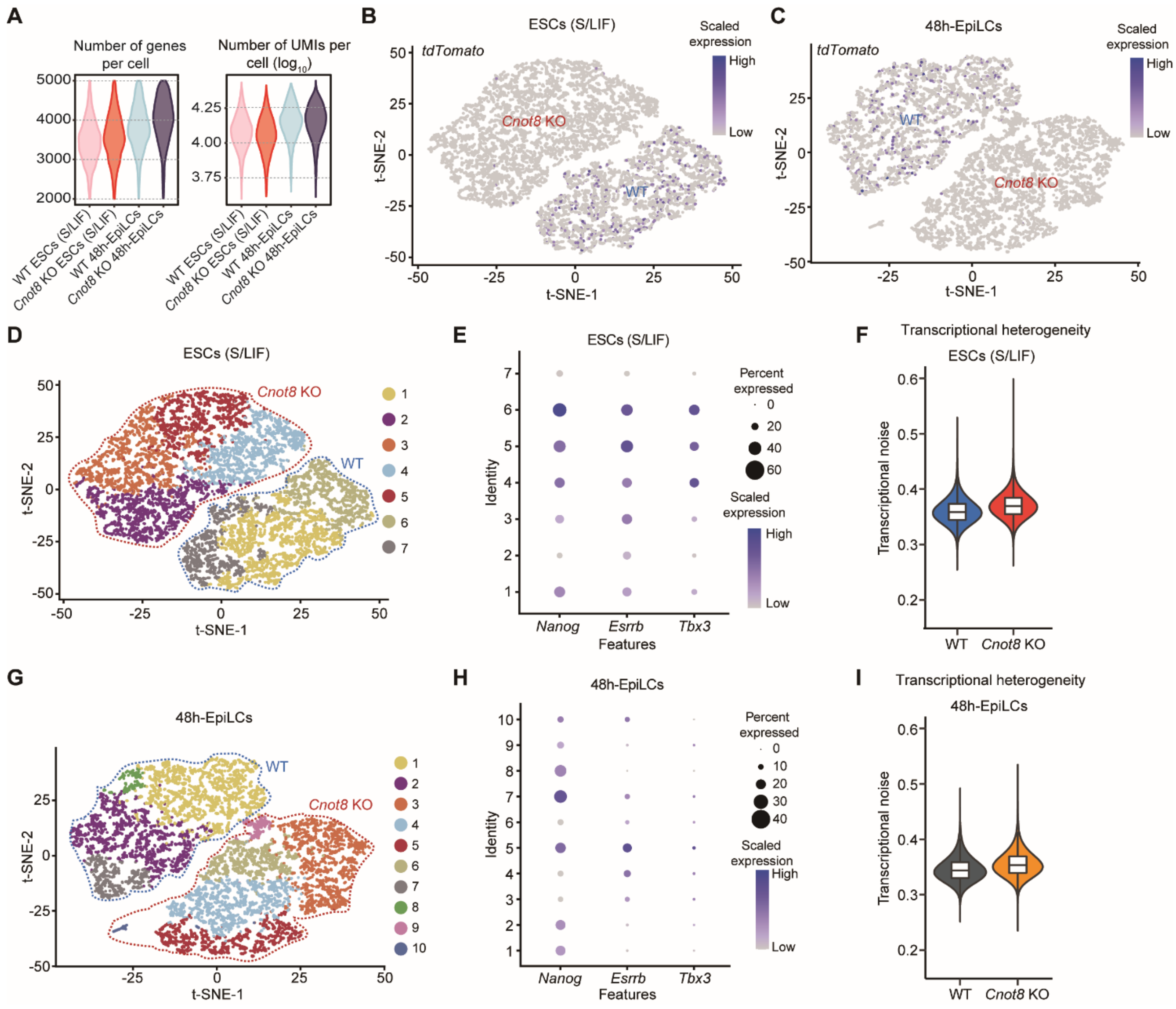

Supplementary Figure S4. scRNA-seq analysis of ESCs (S/LIF) and 48h-EpiLCs.

(**A**) Violin plots indicating the number of detected genes (left) and transcripts (right) in different types of cells. (**B**, **C**) scRNA-seq data of WT and *Cnot8* KO ESCs (S/LIF) (**B**) and 48h-EpiLCs (**C**). The expression of *tdTomato* was shown in WT and *Cnot8* KO cells. (**D**) All cells from WT and *Cnot8* KO ESCs were clustered into 7 cell populations through t-SNE analysis. (**E**) Gene expression level of *Nanog*, *Esrrb*, or *Tbx3* in different clusters of ESCs. The scaled expression levels of genes in each cluster were indicated and the size of a dot represented the percentage of cells expressed with *Nanog*, *Esrrb*, or *Tbx3* in all cells within each cluster for WT and *Cnot8* KO ESCs. (**F**) Violin plots showing transcriptional noise (defined as cell-to-cell transcriptional variability for the 500 most variable genes) in WT and *Cnot8* KO ESCs. Transcriptional noise may be positively associated with their heterogeneity among the cells. (**G**) All cells from WT and *Cnot8* KO 48h-EpiLCs were clustered into 10 cell populations through t-SNE analysis. (**H**) Gene expression level of *Nanog*, *Esrrb*, or *Tbx3* in different clusters. The scaled expression levels of genes in each cluster were indicated and the size of a dot represented the percentage of cells expressed with *Nanog*, *Esrrb*, or *Tbx3* in all cells within each cluster for WT and *Cnot8* KO 48h-EpiLCs. (**I**) Violin plots showing transcriptional noise in WT and *Cnot8* KO 48h-EpiLCs.

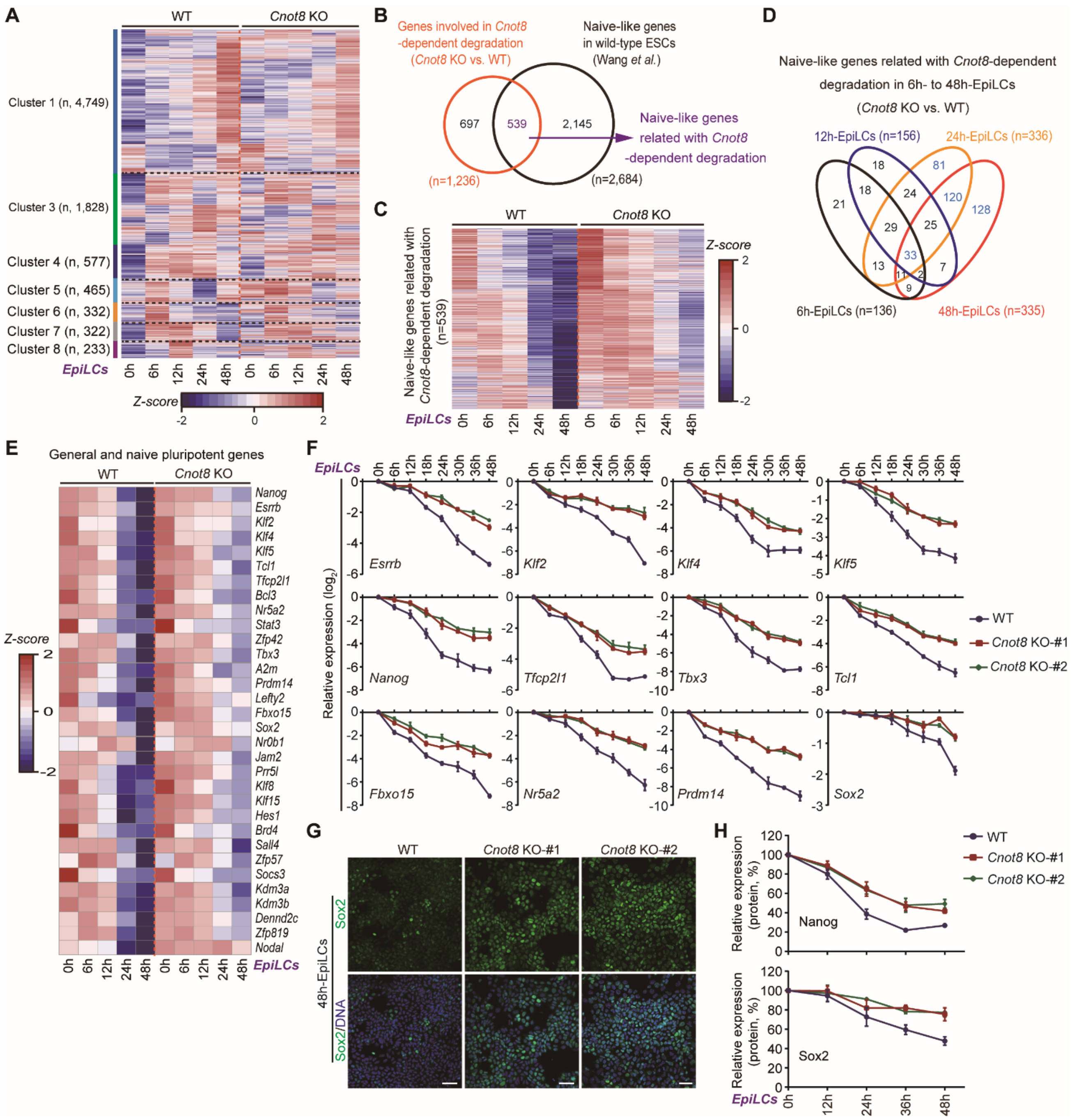

Supplementary Figure S5. Cnot8 regulates naïve gene expression during the differentiation of ESCs. (**A**) Heatmap of the transcript expressions in cluster 1 (n, 4,749), 3 (n, 1,828), 4 (n, 577), 5 (n, 465), 6 (n, 332), 7 (n, 322), and 8 (n, 233) during the differentiation of WT and *Cnot8* KO ESCs. (**B**) Venn diagram showing the overlapped genes between naïve-like genes (n, 2,684) and the genes (n, 1,236) whose mRNA degradation is involved in *Cnot8*. The naïve-like genes were from the previous report (1). The numbers of each class genes were shown. (**C**) Heatmap of the expression of naïve-like genes involved in *Cnot8*-dependent degradation (n, 539) during the differentiation of WT and *Cnot8* KO ESCs into EpiLCs. (**D**) Venn diagram showing intersection of genes involved in *Cnot8*-dependent degradation (n, 539) in 6h- (n, 136), 12h- (n, 156), 24h- (n, 336), and 48h- (n, 335) EpiLCs. The numbers of each class genes were shown in WT and *Cnot8* KO cells. (**E**) Heatmap of the expression of pluripotent genes in WT and *Cnot8* KO ESCs and EpiLCs at specific time points. (**F**) qRT-

PCR analysis for the representative naïve GRN genes in WT and *Cnot8* KO ESCs and EpiLCs at specific time points. The values in ESCs were set as 0. Data were represented as mean ± SEM. n = 3 biological replicates. (**G**) Immunostaining results of Sox2 protein (green) in WT and *Cnot8* KO 48h-EpiLCs. DNA was counterstained with Hoechst 33342 (blue). Scale bar, 50 μm. (**H**) Quantification (for Figure 4D) of Western blot for Nanog and Sox2 proteins in WT and *Cnot8* KO cells during induction of EpiLC differentiation. The levels of protein expression in ESCs were set as 100%. Data were represented as mean ± SEM. Three biological replicates were used for these analyses.

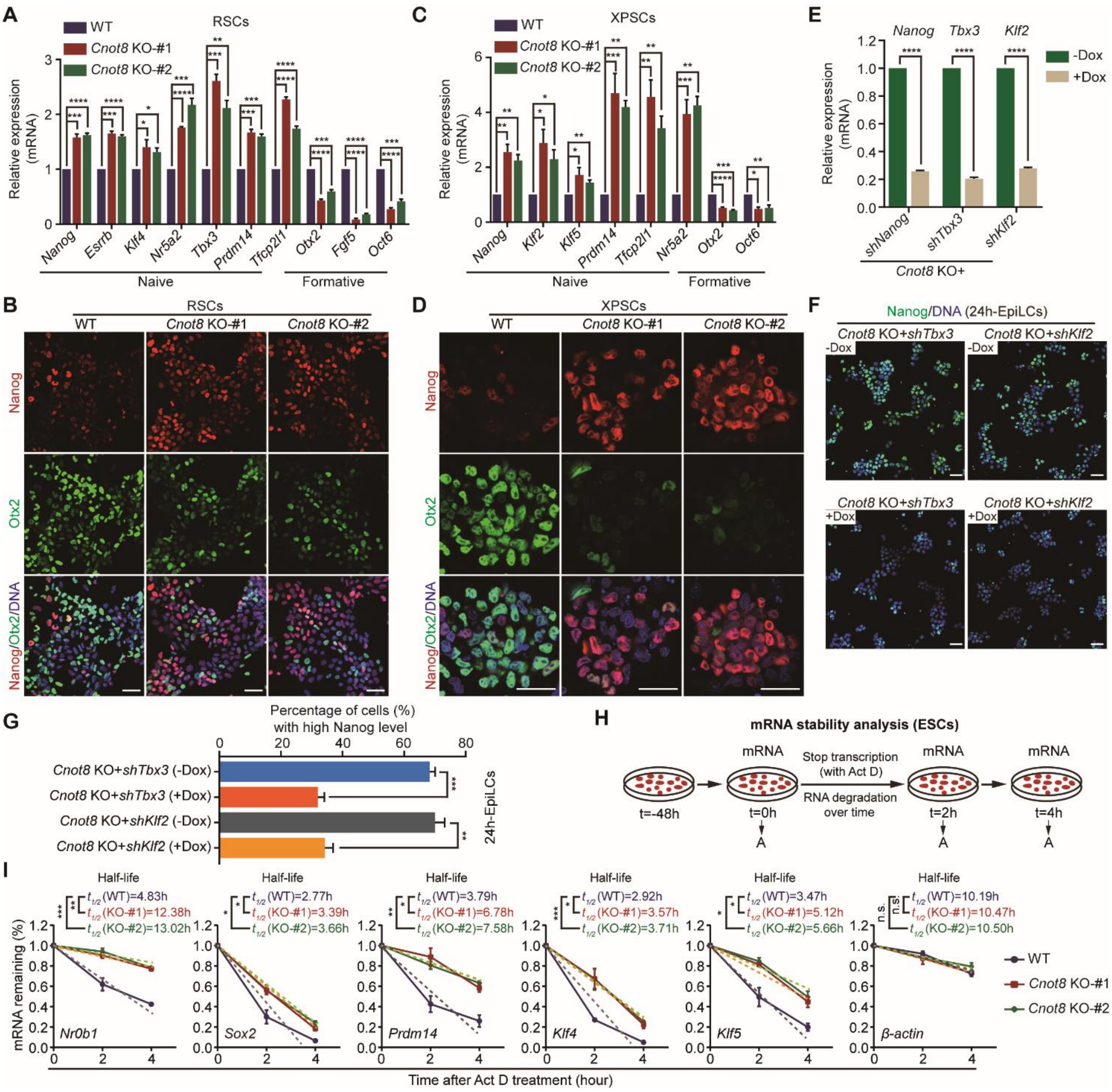

Supplementary Figure S6. Cnot8 regulates gene expression by controlling mRNA stability.

(**A**) qRT-PCR results of representative naïve and formative pluripotent genes in WT and *Cnot8* KO RSCs. Data were represented as mean ± SEM. n = 3 biological replicates. *$\rho < 0.05$, **$\rho < 0.01$, ***$\rho < 0.001$, ****$\rho < 0.0001$ by two-tailed Student's *t* test. (**B**) Immunostaining results of Nanog (red) and Otx2 (green) proteins in WT and *Cnot8* KO RSCs. DNA was counterstained with Hoechst 33342 (blue). Scale bar, 50 μm. (**C**) qRT-PCR results of representative naïve and formative pluripotent genes in WT and *Cnot8* KO XPSCs. Data were represented as mean ± SEM. n = 3 biological replicates. *$\rho < 0.05$, **$\rho < 0.01$, ***$\rho < 0.001$, ****$\rho < 0.0001$ by two-tailed Student's *t* test. (**D**) Immunostaining results of Nanog (red) and Otx2 (green) proteins in WT and *Cnot8* KO XPSCs. DNA was counterstained with Hoechst 33342 (blue). Scale bar, 50 μm. (**E**) qRT-PCR results showing knockdown efficiencies of *Nanog*, *Tbx3*, and *Klf2* mRNAs. *Cnot8* KO ESCs reintroduced with TetON-*shNanog*, TetON-*shTbx3*, or TetON-*shKlf2* were treated with (+) or without (-) Dox (2 μg/ml) for 72 h and examined with qRT-PCR with specific primers for *Nanog*, *Tbx3*, and *Klf2*. Data were represented as mean ± SEM. n = 3

biological replicates. ****$\rho < 0.0001$ by two-tailed Student's *t* test. (**F**) Immunostaining results of Nanog protein (green) in 24h-EpiLCs from *Cnot8* KO ESCs with or without knockdown of *Tbx3* or *Klf2*. DNA was counterstained with Hoechst 33342 (blue). Scale bar, 50 μm. (**G**) Percentage of cells with high level of Nanog protein was analyzed in 24h-EpiLCs from *Cnot8* KO ESCs with or without knockdown of *Tbx3* or *Klf2*. The high Nanog protein referred to the fold change ($Density^{Nanog}/Density^{DNA}$) >= 1 in one cell. Data were represented as mean ± SEM. n = 3 biological replicates. **$\rho < 0.01$, ***$\rho < 0.001$ by two-tailed Student's *t* test. (**H**) A schematic illustration of mRNA stability assay. Act D, Actinomycin D; A, Analysis. (**I**) mRNA stability analysis for representative genes. The expression levels of mRNAs were measured by qRT-PCR after inhibition of transcription at 0, 2, or 4 h after the treatment of actinomycin D. The $t_{1/2}$ value reflected the mRNA half-life of each gene. The dashed lines represented linear regression lines. The values at 0 h were set as 1. Data were represented as mean ± SEM. n = 3 biological replicates. *$\rho < 0.05$, **$\rho < 0.01$, ***$\rho < 0.001$, ns, not significant by two-tailed Student's *t* test.

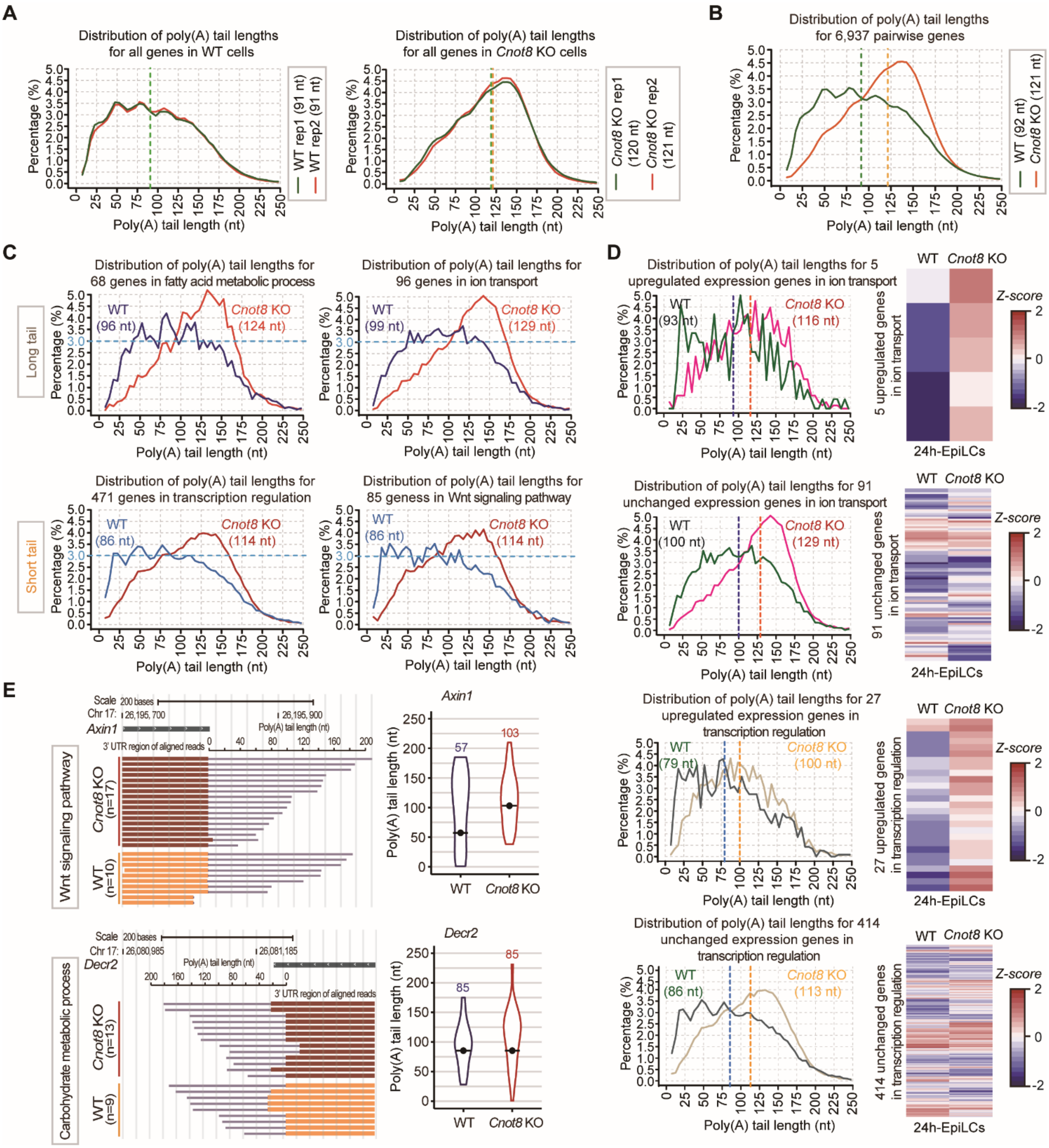

Supplementary Figure S7. Cnot8 regulates the poly(A) tail lengths of mRNAs.

(**A**) PAIso-seq assay showing distribution of poly(A) tail lengths for all genes in each replicate of WT (left) or *Cnot8* KO (right) 24h-EpiLCs. The median poly(A) tail lengths for all genes of each replicate were presented in parentheses and marked by dashed vertical lines. nt, nucleotide. (**B**) PAIso-seq assay showing distribution of poly(A) tail lengths for the pairwise genes in WT and *Cnot8* KO 24h-EpiLCs. The median poly(A) tail lengths for these genes of each genotype were presented in parentheses and marked by dashed vertical lines. (**C**) PAIso-seq assay showing distribution of poly(A) tail lengths for all genes in fatty acid metabolic process (n, 68), ion transport (n, 96), transcription regulation (n, 471), and Wnt signaling pathway (n, 85) in WT and *Cnot8* KO cells.

The median poly(A) tail lengths for these genes of each genotype were presented in parentheses. A light blue dotted line indicated the shifted distribution of the poly(A) tail lengths for these genes in *Cnot8* KO cells. (**D**) Distribution of poly(A) tail lengths and expression levels for the upregulated or unchanged expression genes related with ion transport or transcription regulation in *Cnot8* KO 24h-EpiLCs compared with controls. The upregulated expression genes included some naïve pluripotent genes, such as *Klf2, Tfcp2l1*, *Esrrb*, *Klf5*, *Nanog*, and *Zfp819*, were mainly enriched in transcription regulation. (**E**) Examples of PAIso-seq assay (left) and Violin plots (right) showing poly(A) tails for *Axin1* involved in Wnt signaling pathway and *Decr2* related with carbohydrate metabolic process. Brown (*Cnot8* KO) and orange (WT) bars indicated the 3' UTR regions that were aligned the reads for each transcript. Light blue bars were poly(A) tails of the transcripts. n, number of the transcripts.

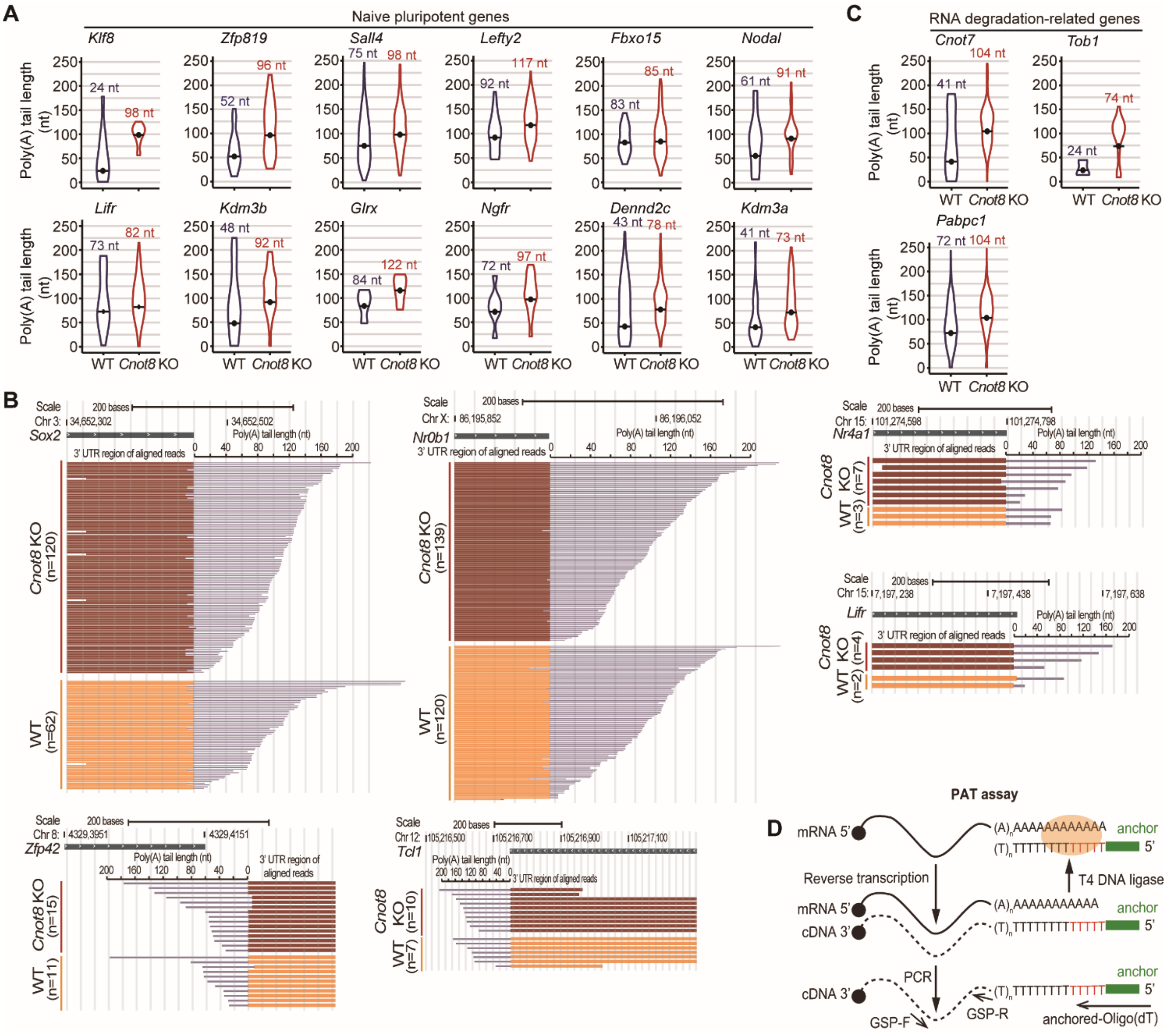

Supplementary Figure S8. Examples of PAIso-seq assay.

(**A**) Violin plots showing poly(A) tail lengths of the mRNAs of representative naïve pluripotent genes in *Cnot8* KO and control cells. The geometric mean length of poly(A) tail for individual gene in each sample was shown with black dots and presented at top. (**B**) Examples of PAIso-seq assay showing poly(A) tails for representative pluripotent gene *Sox2*, *Nr0b1*, *Nr4a1*, *Zfp42*, *Lifr*, and *Tcl1*. Brown (*Cnot8* KO) and orange (WT) bars indicated the 3' UTR regions that were aligned the reads to each transcript. Light blue bars were poly(A) tails of the transcripts. n, number of the transcripts. (**C**) Violin plots showing poly(A) tail lengths for representative RNA degradation-related genes in *Cnot8* KO and control cells. The geometric mean length of poly(A) tail for individual gene in each sample was shown with black dots and presented at top. (**D**) A schematic illustration of the mRNA poly(A) test (PAT) assay. GSP (-F, -R), gene specific primer (F, forward; R, reverse).

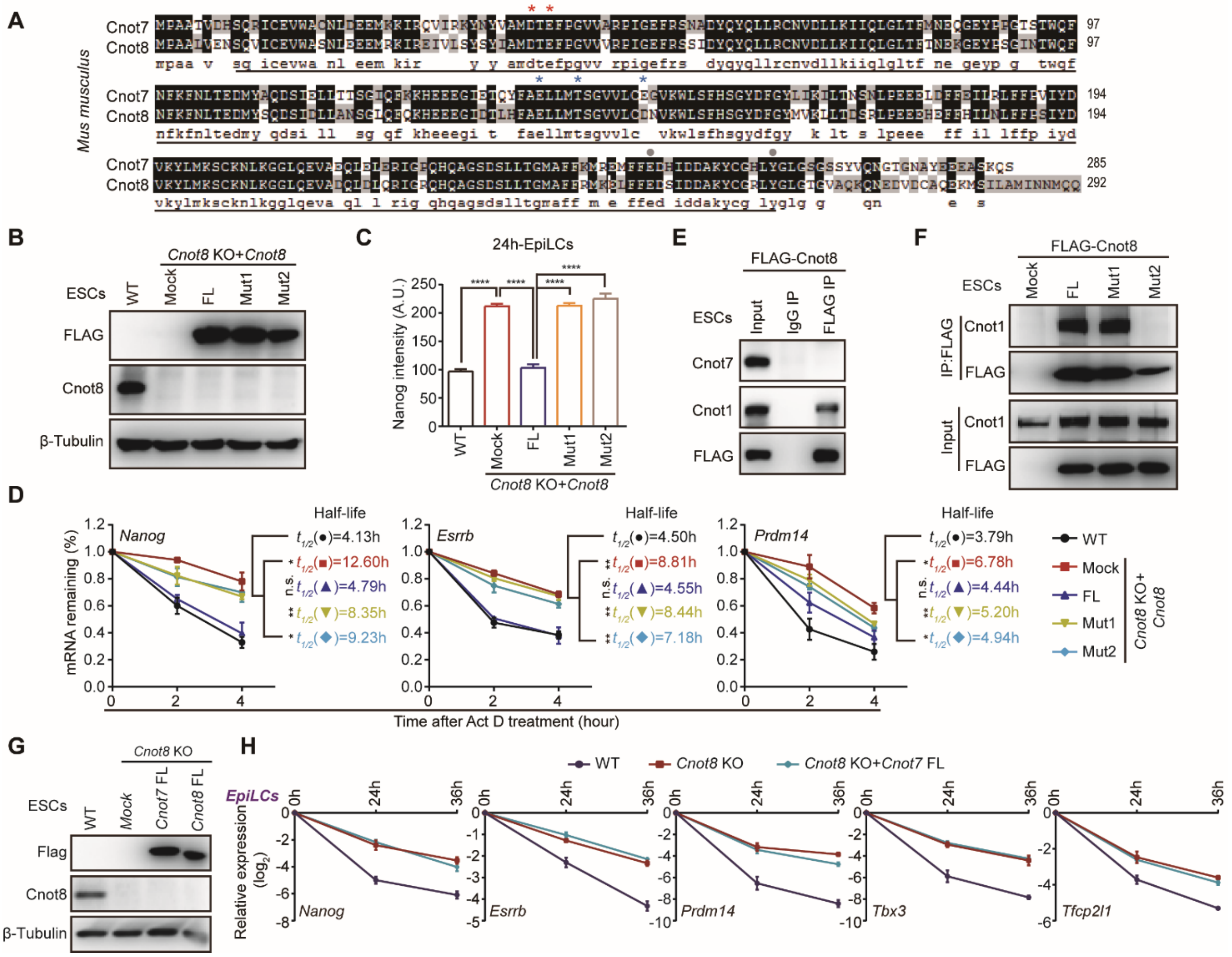

Supplementary Figure S9. Cnot8 regulates mRNA poly(A) tail lengths of naïve GRN genes through its deadenylase activity and Ccr4-Not complex.

(**A**) Protein sequence alignments of *Mus musculus* Cnot7 and Cnot8 orthologs. The conserved residues were highlighted by dark. The conserved DEDD domain was marked with black underline. The two catalytic residues of D40 and E42 were marked with red asterisks. The three residues of E138, T142, and D149 involved in the interaction for Cnot1 were marked with blue asterisks. The two residues involved in the Cnot7/8-Tob1 interaction were labeled by gray circles. (**B**) Western blot analysis of full-length (FL) and two mutant fragments (Mut1 and Mut2) of Cnot8 using anti-FLAG and -Cnot8 antibodies in ESCs. β-Tubulin served as a loading control. Mock, empty vector. (**C**) Quantification of Nanog protein (for Figure 6D) in 24h-EpiLCs from WT and rescued ESCs. Data were represented as mean ± SEM. n = 3 biological replicates. ****$\rho < 0.0001$ by two-tailed Student's *t* test. Rescued ESCs, *Cnot8* KO ESCs overexpressed with empty vector (Mock), Cnot8 FL, Cnot8 Mut1, and Cnot8 Mut2 fragments; A.U., arbitrary unit. (**D**) mRNA stability analysis for representative naïve pluripotent genes. The expression levels of naïve pluripotent genes were measured by qRT-PCR after inhibition of transcription at 0, 2, or 4 h after the treatment of actinomycin D. The $t_{1/2}$ value reflected the mRNA half-life of each gene. The dashed lines represented linear regression lines. The values at 0 h were set as 1. Data were represented as mean ± SEM. n = 3 biological replicates. *$\rho < 0.05$, **$\rho < 0.01$, ns, not significant by two-tailed Student's *t* test. (**E**) Co-IP assay using anti-FLAG antibody in FLAG-Cnot8 overexpressed ESCs. IgG served as a negative control. (**F**)

Co-IP assay of the interaction of FLAG-tagged Cnot8 Mut1 and Mut2 with Cnot1 in ESCs. Mock, ESCs transfected with empty vector. (**G**) Western blot analysis of Cnot7 FL and Cnot8 FL proteins in *Cnot8* KO ESCs. β-Tubulin served as a loading control. Mock, ESCs transfected with empty vector. (**H**) qRT-PCR results showing expression of naïve pluripotent markers in the indicated EpiLCs at different time points. Data were represented as mean ± SEM. n = 3 biological replicates.

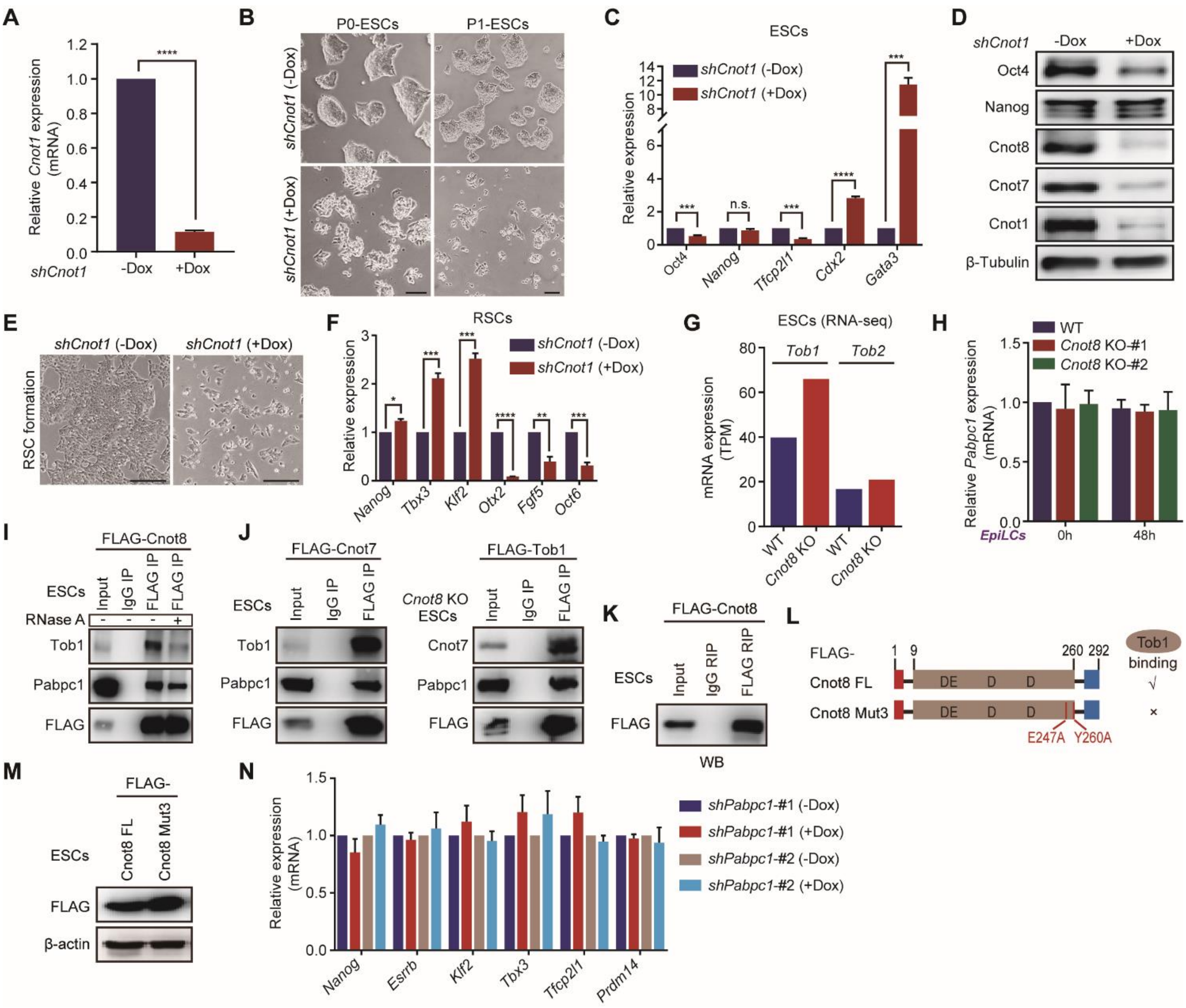

Supplementary Figure S10. Cnot8 interacts with Tob1 and Pabpc1 to regulate mRNA clearance.

(**A**) qRT-PCR results showing knockdown efficiency of *Cnot1* mRNA after WT ESCs ectopically expressed with TetON-*shCnot1* construct were treated with (+) or without (-) Dox (2 μg/ml) for 72 h. Data were represented as mean ± SEM. n = 3 biological replicates. ****ρ < 0.0001 by two-tailed Student's *t* test. (**B**) Morphology of ESCs with or without *Cnot1* knockdown. Scale bar, 50 μm. (**C**) qRT-PCR results showing expression of indicated genes in the ESCs with or without *Cnot1* interfere. Data were represented as mean ± SEM. n = 3 biological replicates. ***ρ < 0.001, ****ρ < 0.0001, ns, not significant by two-tailed student's *t* test. (**D**) Western blot analysis of indicated proteins in the ESCs with or without *Cnot1* knockdown. β-Tubulin served as a loading control. (**E**) Morphology of RSCs differentiated from the ESCs with or without *Cnot1* interfere. Scale bar, 50 μm. (**F**) qRT-PCR results showing expression of naïve and formative pluripotent markers in RSCs differentiated from the ESCs with or without *Cnot1* knockdown. Data were represented as mean ± SEM. n = 3 biological replicates. *ρ < 0.05, **ρ < 0.01, ***ρ < 0.001, ****ρ < 0.0001 by two-tailed student's *t* test. (**G**) RNA-seq results of mouse *Tob1* and *Tob2* mRNAs in WT and *Cnot8* KO ESCs. (**H**) qRT-PCR results of *Pabpc1* mRNA in WT and *Cnot8* KO ESCs and 48h-EpiLCs. Data were represented as mean ± SEM. n = 3 biological replicates. (**I**) Co-IP analysis of the interaction of Cnot8 with Tob1 and Pabpc1 after treatment with or without RNase A. Co-IP was performed in FLAG-Cnot8 overexpressed ESCs. IgG served as a negative control. (**J**) Co-IP and reverse Co-IP analyses

of the interaction between Cnot7 and Tob1/Pabpc1. Co-IP was performed in FLAG-Cnot7 overexpressed ESCs, while reverse Co-IP was conducted in *Cnot8* KO ESCs overexpressed with FLAG-Tob1. IgG served as a negative control. (**K**) Western blot analysis of the products from RIP assay with anti-FLAG antibody. IgG served as a negative control. (**L**) Schematic representation of Cnot8 full-length (FL) protein and mutant fragment (Mut3). The mutant positions of amino acids were indicated. (**M**) Western blot analysis of Cnot8 full-length (FL) and mutant (Mut3) fragments with mouse anti-FLAG antibody in their overexpressed ESCs. β-actin served as a loading control. (**N**) qRT-PCR results showing relative expression levels of representative naïve pluripotent genes after WT ESCs ectopically expressed with TetON-*shPabpc1* were treated with (+) or without (-) Dox (2 μg/ml) for 72 h. Data were represented as mean ± SEM. n = 3 biological replicates.

Supplementary Tables

Supplementary Table S1. Primers used in this study.

**Gene Name**	**Primers (5'-3')**	
Genotyping and sequencing primers		
Cnot6	P1	TCAAATCCAGCAGAACTCGG
	P2	CCAGAGTTTACCGGCAGAAC
	P3	GCCAACATCCCTAACCTCTG
Cnot6-seq-F	Forward	TGTTAAGCCTTCTCAAACCG
Cnot6-seq-R	Reverse	AACTAAGCAATGGACCCACC
Cnot6l	P1	CATCATGGTAGCCAATGGGA
	P2	TTACACCATCATGTCCGCAG
	P3	CAGACAGGGATCCAAATTCTTC
Cnot6l-seq-F	Forward	GCTAATCTTTCACTGTGGTTCC
Cnot6l-seq-R	Reverse	GCTACCCTTGCTCAAGATAATC
Cnot8	P1	GTCATGTTAAGGATTCGGGT
	P2	GTTTTCTACAAGTGCCGCAG
	P3	GTTTTCTACAAGTGCATCCTGAAG
Cnot8-seq-F	Forward	TTCTACCTTGGCCTCCTGAG
Cnot8-seq-R	Reverse	CACACATACTGGGCACAATG
Cnot7	P1	CATGTCTGTGGTAGCCTCTG
	P2	CCACTCCTGAAGTCATAAGAAG
	P3	CAAAGAACCACTCCTGAAGT
Cnot7-seq-F	Forward	TTGTAGTTGTAGGCATAAGTGGAG
Cnot7-seq-R	Reverse	ATAGAAGATCTTTGGTACACGCAC
CRISPR sgRNA sequences		
Cnot6	sgRNA	CGAGTTCTGCCGGTAAACTC
Cnot7	sgRNA	GCAGAACTTCTTATGACTTC
Cnot8	sgRNA	CTGTTTTCTACAAGTGCCGC
Cnot6l	sgRNA1	TTACACCATCATGTCCGCAG
	sgRNA2	CCAATGGGAAAAAATCTCAC
shRNA primers		
shCnot1	Forward	CTAGCTGTTAGAGGCTTACGTTAAAGTACTAGTCTTTAACGTAAGCC TCTAACATTTTTG
	Reverse	AATTCAAAAATGTTAGAGGCTTACGTTAAAGACTAGTACTTTAACGT AAGCCTCTAACAG
shNanog	Forward	CTAGCCCTGAGCTATAAGCAGGTTAATACTAGTTTAACCTGCTTATA GCTCAGGTTTTTG
	Reverse	AATTCAAAAACCTGAGCTATAAGCAGGTTAAACTAGTATTAACCTGC TTATAGCTCAGGG
shTbx3	Forward	CTAGCGAGCCAACGATATCCTGAATACTAGTTTCAGGATATCGTTG GCTCTTTTTG
	Reverse	AATTCAAAAAGAGCCAACGATATCCTGAAACTAGTATTCAGGATATC GTTGGCTCG
shKlf2	Forward	CTAGCGGCAAGACCTACACCAAGATACTAGTTCTTGGTGTAGGTCT TGCC TTTTTG
	Reverse	AATTCAAAAAGGCAAGACCTACACCAAGAACTAGTATCTTGGTGTA GGTCTTGCCG
shPabpc1-#1	Forward	CTAGCCCTAGCCAAATTGCTCAACTATACTAGTTAGTTGAGCAATTT GGCTAGGTTTTTG
	Reverse	AATTCAAAAACCTAGCCAAATTGCTCAACTAACTAGTATAGTTGAGC AATTTGGCTAGGG
shPabpc1-#2	Forward	CTAGCCCATCGACAATAAAGCACTATTACTAGTATAGTGCTTTATTG TCGATGGTTTTTG
	Reverse	AATTCAAAAACCATCGACAATAAAGCACTATACTAGTAATAGTGCTT

		TATTGTCGATGGG
PAIso-seq library constructing primers		
TSO	Forward	AAGCAGTGGTATCAACGCAGAGTACATrGrG+G
RT-P	Reverse	AAGCAGTGGTATCAACGCAGAGTAC
BC1	Forward	TGCTATCTGAGATACT
BC2	Forward	GAGTCTCGATATACTA
BC3-2dU-P	Reverse	AAGCAGTGGTATCAACGCAGAGTACTACTAGAGTAGCACTCdUTTTT TTTTdUTTTTTTTT
PCR-P	Forward	AAGCAGTGGTATCAACGCAGAGT
qRT-PCR primers		
Oct4	Forward	TGAGAACCTTCAGGAGATATGCAA
	Reverse	CTCAATGCTAGTTCGCTTTCTCTTC
Sox2	Forward	CAGGAGAACCCCAAGATGCACAA
	Reverse	AATCCGGGTGCTCCTTCATGTG
Nanog	Forward	CAGAAAAACCAGTGGTTGAAGACTAG
	Reverse	GCAATGGATGCTGGGATACTC
Prdm14	Forward	ACAGCCAAGCAATTTGCACTAC
	Reverse	TTACCTGGCATTTTCATTGCTC
Nr5a2	Forward	GGGAAGGAAGGGACAATCTT
	Reverse	GCGAGACTCAGGAGGTTGTT
Klf5	Forward	TACGGGCGAGAAGCCCTACA
	Reverse	GGCACACCATGCACTGGAAC
Tcl1	Forward	CTCCATGTATTGGCAGATCCTGTA
	Reverse	CTCCGAGTCTATCAGTTCAAGCAA
Esrrb	Forward	TTTCTGGAACCCATGGAGAG
	Reverse	AGCCAGCACCTCCTTCTACA
Tfcp2l1	Forward	AGGTGCTGACCTCCTGAAGA
	Reverse	GTTTTGCTCCAGCTCCTGAC
Klf2	Forward	CGCACCTAAAGGCGCATCTG
	Reverse	TTCGGTAGTGGCGGGTAAGC
Nr0b1	Forward	TCCAGGCCATCAAGAGTTTC
	Reverse	ATCTGCTGGGTTCTCCACTG
Fbxo15	Forward	TCACGTTGGAAAGCTACTTCAGTG
	Reverse	CTGTTGGTGAGGGCTGCTTTCA
Tbx3	Forward	TCTCCATCGTGGGGACATC
	Reverse	CGTGCTCCTCCTTGCTCTC
Klf4	Forward	TGCTCCCGTCCTTCTCCAC
	Reverse	CCTCACGCCAACGGTTAGTC
Dnmt3b	Forward	CTCGCAAGGTGTGGGCTTTTGTAAC
	Reverse	CTGGGCATCTGTCATCTTTGCACC
Fgf5	Forward	GAAATATTTGCTGTGTCTCAGGG
	Reverse	TAAATTTGGCACTTGCATGG
Otx2	Forward	GACCCGGTACCCAGACATC
	Reverse	GCTCTTCGATTCTTAAACCATACC
Oct6	Forward	CCCACTGCCTGCTCCCTAT
	Reverse	GGAATCCCTAACCCCGCAC
Cdx2	Forward	TCCTGCTGACTGCTTTCTGA
	Reverse	CCCTTCCTGATTTGTGGAGA
Gata3	Forward	CGGGTTCGGATGTAAGTCGA
	Reverse	GTAGAGGTTGCCCCGCAGT
Gata4	Forward	CCGAGGGTGAGCCTGTATGT
	Reverse	AGGACCTGCTGGCGTCTTAG
Gata6	Forward	TTAACACTGATTGCTGCAACG
	Reverse	GTTCATCGTAACGTGGCTGA
Sox7	Forward	AGATGCTGGGAAAGTCATGG

	Reverse	GCTTGCCTTGTTTCTTCCTG
Tob1	Forward	AAGTTCGGCTCCACCAAAATGAAG
	Reverse	CTGGGATGATGACGGCTGCTG
Pabpc1	Forward	TGCAGAGGATGGCAAGTGTACG
	Reverse	GCTAGGAGGATAGTATGCAGCAC
β-actin	Forward	ATGCTCCCCGGGCTGTAT
	Reverse	CATAGGAGTCCTTCTGACCCATTC
Gapdh	Forward	CCCCAATGTGTCCGTCGTG
	Reverse	TGCCTGCTTCACCACCTTCT
Cnot6	Forward	TGGAAAGAAATCACATTGGGCAG
	Reverse	TGGCAATGTCTGAAGGAATACAGGA
Cnot6l	Forward	AACCTTGCTCGCATTCCACCTGAT
	Reverse	CGGGTTCCATCTGGGTCCTGGTAT
Cnot8	Forward	CCCGTCCATTTACGATGTGAAATAC
	Reverse	ACTTGGCATCGTCAATACTGTCCTC
Cnot8-KE	Forward	AAATCAAACAGAAGGAGGCTTG
	Reverse	GTTTTCTACAAGTGCCGCAG
Cnot7	Forward	GGTGGATTACAGGAAGTTGCTG
	Reverse	GGATGAGCCAGAACCAAGG
Cnot7-KE	Forward	GCTCGGACTGACCTTTATGAATGA
	Reverse	CAAAGAACCACTCCTGAAGTCATAA
Cnot1	Forward	CTATCCTCAGTATTGTCAGCACCTG
	Reverse	CTGAGCCAGTGCAATACTTCCAG
RT-PCR primers		
Nanog	Forward	ATGAAGTGCAAGCGGTGGCAGAAA
	Reverse	CCTGGTGGAGTCACAGAGTAGTTC
Sox2	Forward	GGCGGCAACCAGAAGAACAG
	Reverse	GCTTGGCCTCGTCGATGAAC
Tcl1	Forward	CACCATGGCTACCCAGCGGGCACACA
	Reverse	TTATTCATCGTTGGACTCCGAGTCTATCAG
Nr0b1	Forward	TCCAGGCCATCAAGAGTTTC
	Reverse	ATCTGCTGGGTTCTCCACTG
Tfcp2l1	Forward	GTGAAGCTACATGAAGAGACCTTAAC
	Reverse	GAATATCAATGTCCAGGATGC
Esrrb	Forward	GTTGCGGCTCCTTCATCAAG
	Reverse	GTTAAGCATGTACTCGCATTTGATG
Klf4	Forward	GAAGGGAGAAGACACTGCGT
	Reverse	GATAAAGTCTAGGTCCAGGAGGTC
Klf5	Forward	GAGCTGGTCCAGACAAGATGTG
	Reverse	TGGGAGCTGAAGATACTAGTGAACTC
Tbx3	Forward	CGAAGAAGACGTAGAAGATGAC
	Reverse	GTATATACATTCTCTTTGGCATTTC
Prdm14	Forward	GTCACCATGGAACCGAATTC
	Reverse	TGGGACGACTTAATTCCATCAG
Fbxo15	Forward	ACGTTGGAAAGCTACTTCAGTG
	Reverse	CTCTTAAGATGATTGTCCAACC
Klf2	Forward	CACACATACTTGCAGCTACACCAAC
	Reverse	GACCTGTGTGCTTTCGGTAGTG
Hdac11	Forward	CAACGAGCTGAAGTGGTCCTTTG
	Reverse	GAAGCCACCACCAACATTGAT
Vector cloning primers		
FLAG- EGFP-Cnot8 FL	Forward	TAATCTAGAATGCCTGCGGCACTTGTAGAAAACAGTCAGGTCATC
	Reverse	TTAGGATCCTCACTGCTGCATGTTGTTGATCATGGCCAAGATG
FLAG-EGFP-Cnot8 Mut1	Forward	CTATATCGCTATGGCCACAGCCTTCCCAGGTGTTGTTGTAC
	Reverse	GTACAACAACACCTGGGAAGGCTGTGGCCATAGCGATATAG

FLAG-EGFP-Cnot8 Mut2	Forward	CACTTTGCAAAGCTGCTTATGTACTCGGGAGTGGTTCTCTGTAAGAACGTC
	Reverse	GACGTTCTTACAGAGAACCACTCCCGAGTACATAAGCAGCTTTGCAAAG
FLAG-EGFP-Cnot8 Mut3	Forward	CTATTCTTTGCCGACAGTATTGACGATGCCAAGTATTGTGGGCGCCTCGCCGGCCTG
	Reverse	GCCGGCGAGGCGCCCACAATACTTGGCATCGTCAATACTGTCGGCAAAGAATAG
FLAG-EGFP-Cnot7 FL	Forward	TAATCTAGAATGCCAGCAGCAACCGTAGATCATAGCCAAAGAATTTGTGAAG
	Reverse	TTAGGATCCTCATGACTGCTTGCTGGCTTCCTCTTCATATGCATTCCCTG
FLAG-EGFP-Tob1	Forward	TAATCTAGAATGCAGCTTGAAATCCAAGTAGCACTC
	Reverse	TTAGGATCCTTAGTTAGCCATAACAGGCTGGAATTGCTG
PAT assay primers		
PAT-P	Reverse	GCGAGCTCCGCGGCCGCGTTTTTTTTTTTT
Klf4	Forward	CAGTCTGTTATGCACTGTGGTTTCAGATG
	Reverse (A0)	GTCGTTCAGATAAAATATTATAGGTTTATTTAAAACTTATTTCTCACCTTGAGTATGC
Tbx3	Forward	CATCTTTATGGTGTTTTGGATTGGTGTACTGAGAG
	Reverse (A0)	CATTTCAATAAAAATTTATTGAAATTTCAGTGA
Tfcp2l1	Forward	GACTGGTCAAATATGTGTGG
	Reverse (A0)	GGGTTTAGTACAAATGGTTTTATTTGCATTCTGACC
Fbxo15	Forward	TGAGTTAGCAGTAGGGAGTCTTGTTATTAGTAAGCTG
	Reverse (A0)	TACCACAAATATGCTTTATTTTAACTTTC
Tcl1	Forward	GAGATGCCAATAAATGAGGGAGAGAAGACTG
	Reverse (A0)	AGTTCTACAAAGCTTTATTAAATCCAGCCGTATC
Prdm14	Forward	TACCACAGCCAAGCAATTTGCACTACCTAGAG
	Reverse (A0)	AAAACCAGAGTAGCATAAGTGTTTATTTTCAGCCTGTC
Nanog	Forward	GGAGTTTGAGGGTAGCTCAG
	Reverse (A0)	AAAGGTAAAATTAGTGTTTTATTTTGTTTAAACTAGTCCAGCTGGCATCGG
Sox2	Forward	CAGGCTGCCGAGAATCCATGTATATATTTGAACTAATACCATCC
	Reverse (A0)	CAGTGTCCATATTTCAAAAATTTATTTATCTCAAACTG
Esrrb	Forward	GAACAGCACCAAAGAGAAGCACTATGTGGAGAG
	Reverse (A0)	CAGCAGACCTTTCCTTATTTATTAGATTTCAGATACATGG
Gapdh	Forward	CAAGGACACTGAGCAAGAGAGGCCCTATC
	Reverse (A0)	GTGGGTGCAGCGAACTTTATTGATGGTATTCAAGAGAG

Supplementary Table S2. Antibody information.

Antibodies	Source	Identifier	Dilution
Goat anti-Oct4	Santa Cruz Biotechnology	sc8628	1:200 (IF); 1:1000 (IB)
Rabbit anti-Nanog	Abcam	ab80892	1:200 (IF); 1:1000 (IB)
Mouse anti-Sox2	Cell signaling Technology	4900	1:200 (IF); 1:1000 (IB)
Rabbit anti-Ezrin	Abcam	ab76247	1:200 (IF)
Goat anti-Otx2	AF1979	R&D systems	1:200 (IF); 1:1000 (IB)
Rabbit anti-Cnot8	Abclonal	A8058	1:1000 (IB)
Mouse anti-Cnot7	Sigma-Aldrich	WH0029883M1	1:1000 (IB)
Rabbit anti-Cnot1	Abclonal	A5969	1:1000 (IB)
Rabbit anti-Pabpc1	Abcam	ab21060	1:200 (IF); 1:1000 (IB)
Rabbit anti-Tob1	Proteintech	14915-1-AP	1:500 (IB)
Rabbit anti-Cleaved Caspase-3	Cell signaling Technology	9661	1:200 (IF)
Mouse anti-FLAG	Sigma-Aldrich	F1804	1:200 (IF); 1:2000 (IB)
Mouse anti-FLAG	Abclonal	AE005	1:2000 (IB)
Mouse anti-Gapdh	Yeasen	30201ES60	1:2000 (IB)
Mouse anti-β-Tubulin	Yeasen	30301ES60	1:2000 (IB)
Mouse anti-β-actin	Yeasen	30101ES50	1:2000 (IB)
Hoechst 33342	Sigma-Aldrich	B2261	1:200 (IF)
Alexa Fluor® 488 AffiniPure Donkey anti-Goat IgG (H+L)	Jackson ImmunoReseach	705-545-003	1:200 (IF)
Alexa Fluor® 594 AffiniPure Donkey anti-Mouse IgG (H+L)	Jackson ImmunoReseach	715-585-150	1:200 (IF)
Alexa Fluor® 488 AffiniPure Donkey anti-Mouse IgG (H+L)	Jackson ImmunoReseach	715-545-150	1:200 (IF)
Alexa Fluor® 488 AffiniPure Donkey anti-Rabbit IgG (H+L)	Jackson ImmunoReseach	711-545-152	1:200 (IF)
Alexa Fluor® 594 AffiniPure Donkey anti-Rabbit IgG (H+L)	Jackson ImmunoReseach	711-585-152	1:200 (IF)
Cy™5 AffiniPure Donkey anti-Rabbit IgG (H+L)	Jackson ImmunoReseach	711-175-152	1:200 (IF)
Alexa Fluor® 647 AffiniPure Donkey anti-Mouse IgG (H+L)	Jackson ImmunoReseach	715-605-151	1:200 (IF)
HRP-labeled Goat anti-Mouse IgG (H+L)	Jackson ImmunoReseach	115-035-062	1:3000 (IB)
HRP-labeled Goat anti-Rabbit IgG (H+L)	Jackson ImmunoReseach	111-035-003	1:3000 (IB)
HRP-labeled Rabbit anti-Goat IgG (H+L)	Jackson ImmunoReseach	305-035-003	1:3000 (IB)

Other Supplementary Information for this manuscript includes the following:

Supplementary Table S3 to S8 as Excel files

Supplementary Table S3. Upregulated and downregulated expression genes in *Cnot7* KO ESCs

Supplementary Table S4. Upregulated and downregulated expression genes in *Cnot8* KO ESCs and EpiLCs

Supplementary Table S5. Genes involved in *Cnot8*-dependent degradation

Supplementary Table S6. Naïve-like genes related with *Cnot8*-dependent degradation

Supplementary Table S7. *Cnot8*-dependent degradation naïve GRN genes

Supplementary Table S8. The list of all GO terms that possess more than 50 genes detected by PAIso-seq

1. Wang, X., Xiang, Y., Yu, Y., Wang, R., Zhang, Y., Xu, Q., Sun, H., Zhao, Z.A., Jiang, X., Wang, X. *et al.* (2021) Formative pluripotent stem cells show features of epiblast cells poised for gastrulation. *Cell Res*, **31**, 526-541.
