# Supplementary material for: Cnot8 eliminates naïve regulation networks and is essential for naïve-to-formative pluripotency transition
Source: Nucleic Acids Res. 2022 Apr 7;50(8):4414–35. doi: 10.1093/nar/gkac236 (PMC9071485; doi:10.1093/nar/gkac236)
Supplement: gkac236_Supplemental_Files [file gkac236_supplemental_files.zip › Supplementary Information.docx]

**Supplementary Information**

**Supplementary Figures**

**Supplementary Figure S1.** *Cnot8* is essential for mouse early embryonic development

**Supplementary Figure S2.** Derivation and maintenance of *Cnot7* or *Cnot8* KO mESCs

**Supplementary Figure S3.** *Cnot8* is required for the differentiation of naïve ESCs into formative state.

**Supplementary Figure S4.** scRNA-seq analysis of ESCs (S/LIF) and 48h-EpiLCs

**Supplementary Figure S5.** Cnot8 regulates naïve gene expression during the differentiation of ESCs.

**Supplementary Figure S6.** Cnot8 regulates gene expression by controlling mRNA stability

**Supplementary Figure S7.** Cnot8 regulates the poly(A) tail lengths of mRNAs

**Supplementary Figure S8.** Examples of PAIso-seq assay

**Supplementary Figure S9.** Cnot8 regulates mRNA poly(A) tail lengths of naïve GRN genes through its deadenylase activity and Ccr4-Not complex

**Supplementary Figure S10.** Cnot8 interacts with Tob1 and Pabpc1 to regulate mRNA clearance

**Supplementary Tables**

**Supplementary Tables S1 and S2 as PDF files**

**Supplementary Table S1.** Primers used in this study

**Supplementary Table S2.** Antibody information

**Supplementary Tables S3 to S8 as Excel files**

**Supplementary Table S3.** Upregulated and downregulated expression genes in *Cnot7* KO ESCs

**Supplementary Table S4.** Upregulated and downregulated expression genes in *Cnot8* KO ESCs and EpiLCs

**Supplementary Table S5.** Genes involved in *Cnot8*-dependent degradation

**Supplementary Table S6.** Naïve-like genes involved in *Cnot8*-dependent degradation

**Supplementary Table S7.** *Cnot8*-dependent degradation naïve GRN genes

**Supplementary Table S8.** The list of all GO terms that possess more than 50 genes detected by PAIso-seq
